# The vacuolar H+ ATPase is a novel therapeutic target for glioblastoma

**DOI:** 10.18632/oncotarget.4239

**Published:** 2015-05-22

**Authors:** Andrea Di Cristofori, Stefano Ferrero, Irene Bertolini, Gabriella Gaudioso, Maria Veronica Russo, Valeria Berno, Marco Vanini, Marco Locatelli, Mario Zavanone, Paolo Rampini, Thomas Vaccari, Manuela Caroli, Valentina Vaira

**Affiliations:** ^1^ Department of Pathophysiology and Organ Transplantation, University of Milan, Milan, Italy; ^2^ Division of Neurosurgery, Fondazione IRCCS Ca' Granda, Ospedale Maggiore Policlinico, Milan, Italy; ^3^ Division of Pathology, Fondazione IRCCS Ca' Granda, Ospedale Maggiore Policlinico, Milan, Italy; ^4^ Department of Biomedical, Surgical and Dental Sciences, University of Milan, Milan, Italy; ^5^ Istituto Nazionale Genetica Molecolare “Romeo ed Enrica Invernizzi”, Milan, Italy; ^6^ Surgical Pathology Unit, St. Anna Hospital, Como, Italy; ^7^ IFOM - The FIRC Institute of Molecular Oncology, Milan, Italy

**Keywords:** glioblastoma, vacuolar H+-ATPase, bafilomycin A1, cancer stem cells, organotypic tissue cultures

## Abstract

The vacuolar H^+^ ATPase (V-ATPase) is a proton pump responsible for acidification of cellular microenvironments, an activity exploited by tumors to survive, proliferate and resist to therapy. Despite few observations, the role of V-ATPase in human tumorigenesis remains unclear.

We investigated the expression of ATP6V0C, ATP6V0A2, encoding two subunits belonging to the V-ATPase V0 sector and ATP6V1C, ATP6V1G1, ATPT6V1G2, ATP6V1G3, which are part of the V1 sector, in series of adult gliomas and in cancer stem cell-enriched neurospheres isolated from glioblastoma (GBM) patients. ATP6V1G1 expression resulted significantly upregulated in tissues of patients with GBM and correlated with shorter patients' overall survival independent of clinical variables.

ATP6V1G1 knockdown in GBM neurospheres hampered sphere-forming ability, induced cell death, and decreased matrix invasion, a phenotype not observed in GBM monolayer cultures. Treating GBM organotypic cultures or neurospheres with the selective V-ATPase inhibitor bafilomycin A1 reproduced the effects of ATP6V1G1 siRNA and strongly suppressed expression of the stem cell markers Nestin, CD133 and transcription factors SALL2 and POU3F2 in neurospheres.

These data point to ATP6V1G1 as a novel marker of poor prognosis in GBM patients and identify V-ATPase inhibition as an innovative therapeutic strategy for GBM.

## INTRODUCTION

V-ATPase is a ubiquitously expressed and evolutionary conserved multimeric proton pump that acidifies intracellular compartments and the extracellular milieu. V-ATPase is composed of a membrane-spanning V0 sector regulating proton permeability and a V1 sector providing ATPase activity for V0 rotation relative to membranes. Pumping efficiency is modulated by regulated assembly of the two sectors and by coupling of ATPase activity with V0 rotation [1]. Multiple roles have been attributed to V-ATPase in physiological conditions, among which regulation of pH balance, control of neurotransmitter trafficking and degradation of membrane-bound proteins are the most important [1, 2]. In addition, recent evidence indicated that V-ATPase acts directly in the endolysosomal compartment to modulate signaling pathways important for development that are dysregulated in cancer, such as Notch, Wnt or mTOR signaling [3-7]. This evidence, together with the fact that a variety of human tumors display overexpression of V-ATPase subunits, suggests that V-ATPase might play a crucial, albeit largely unexplored, role in supporting tumorigenesis. So far, overexpression of V-ATPase in human tumors has been associated to invasive and chemoresistant phenotypes [8, 9] and to increased proliferative activity [10, 11, 12]. Cancer cells are characterized by an abnormal basic intracellular microenvironment, and such pH imbalance has been proposed to sustain survival by limiting apoptosis, ultimately enhancing cancer proliferation [11]. Thus, V-ATPase could in principle represent a novel target for anti-neoplastic approaches [13-16].

Gliomas are the most frequent primary brain cancers in adults characterized by poor prognosis and limited therapeutic options. Temozolomide, an alkylating agent representing the standard of care, is not curative and overall mortality rates are high [17, 18]. To date, histologic classification is the most significant prognostic factor in predicting patient survival. Molecular features, including methylation of O6-methylguanine-DNA methyltransferase (MGMT) promoter, isocitrate dehydrogenase 1 or 2 (IDH1/2) gene mutations or 1p/19q chromosomal loci deletion have recently provided additional information. Nevertheless, limited improvement in prognosis has been achieved, especially in adult patients or for high grades tumors [19, 20].

Dysregulated ion balance in glioma stem cells is emerging as a key factor that could contribute to rapid and aggressive tumor formation and relapse [18]. In line with this evidence, increased expression and/or activity of V-ATPase, or of other ion transporters is observed in almost all cancers [11, 15, 18, 21-23]. Therefore understanding of oncogenic mechanisms depending on selective ion transport could lead to more promising and tailored therapeutic approaches. On these premises, the goal of this study was to determine whether V-ATPase is dysregulated in glioma stem cells and to assess whether V-ATPase targeting could represent an innovative strategy for GBM therapy.

## RESULTS

### V-ATPase subunit G1 is up-regulated in human gliomas and in GBM neurospheres

To get insights on the involvement of V-ATPase dysregulation in human gliomas, we evaluated a panel of V-ATPase subunits belonging to either the V0 (ATP6V0C, ATP6V0A2) or the V1 (ATP6V1C, ATP6V1G1, ATPT6V1G2, ATP6V1G3) sectors (Supplementary Figure 1) using Oncomine, a web-based repository of publicly available series of glioma patients. This analysis showed that ATP6V1G1 and ATP6V0A2 are frequently over-expressed in gliomas compared to non-neoplastic brain tissues without concomitant up-regulation of other subunits (Supplementary Figure 2A, 2B). Available brain cancer series were also investigated for V-ATPase gene amplification. Significant copy number gain for ATP6V1G1 gene located at 9q32 was detected only in recurrent GBMs and not in primary tumors, possibly suggesting that gene amplification might be a later event secondary to gene overexpression (Supplementary Figure 2C).

**Figure 1:**
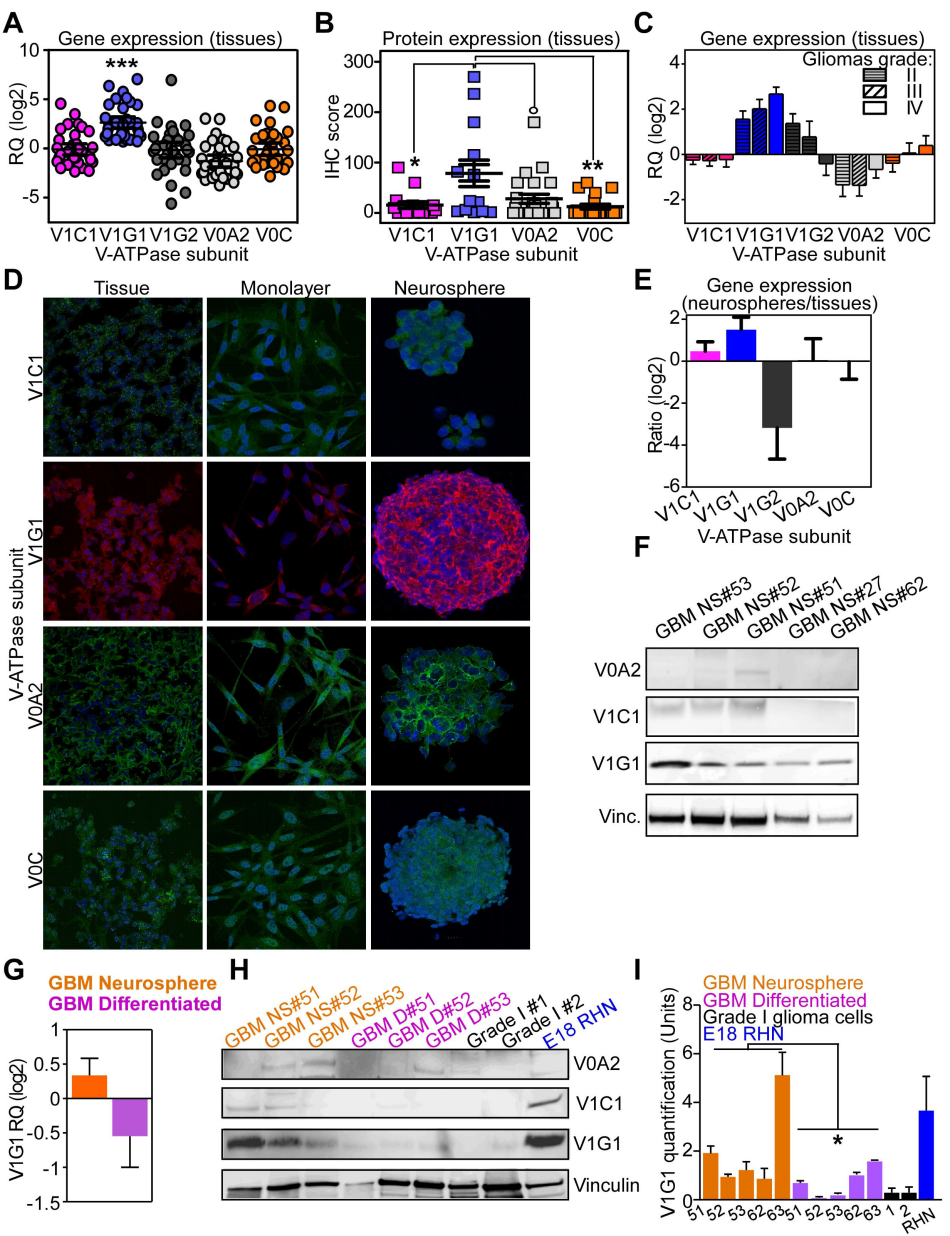
V-ATPase expression in normal brain or gliomas **A.**, **B.** The vacuolar proton pump subunits V1C1, V1G1, V1G2, V0A2 and V0C were analyzed by qPCR (**A.**, RQ) or immunohistochemistry (**B.**, IHC score) in a panel of human gliomas (*n* = 54). °, *p* = 0.03; *, *p* = 0.02; **, *p* = 0.002; ***p < 0.0001 by unpaired *t* tests. Line, mean with SEM. RQ, mRNA relative quantity. **C.** V-ATPase subunits expression was investigated in human glioma tissues classified according to WHO grade II, III or IV (GBM). **D.** Expression of the indicated V-ATPase subunit was analyzed by immunofluorescence in primary glioma tissues and derived neurosphere or differentiated (monolayer) cell cultures from seven individual patients. Representative images are shown. **E.** Glioblastoma neurospheres obtained from individual patients (*n* = 7) were analyzed for differential gene expression of V-ATPase subunits compared to their original tumor tissues. Bars, mean±SD. **F.** GBM neurospheres obtained from 5 individual patients were analyzed for V-ATPase subunit V0A2, V1C1 and V1G1 protein presence by western blotting. Vinculin (Vinc.) was a loading control. **G.** Quantification by qRT-PCR of ATP6V1G1 mRNA level in GBM neurospheres or differentiated cultures obtained as in Supplementary Figure 4B (*n* = 5 patients). **H.** V-ATPase G1 protein expression was analyzed by western blotting in matched GBM neurospheres and differentiated cell monolayers, cultures obtained from Grade I brain tumors (GradeI#1 and #2), and embryonic rat hippocampal neural cells (E18 RHN). Vinculin (vinc.) was a loading control. **I.** Quantification of V-ATPase G1 protein expression obtained as in H was performed with Image J. *, *p* = 0.03 by Wilcoxon signed rank test comparing matched neurosphere and differentiated cultures.

To confirm these *in silico* findings in an experimental set-up, we assayed V-ATPase expression at gene or protein expression levels in a small cohort of brain tumors (Gene expression series; Table 1). Among the V-ATPase subunits analyzed, ATP6V1G1 (V-ATPase G1) was the most expressed in human gliomas (Figure 1A and 1B), whereas expression of the V-ATPase G1 paralog ATP6V1G3 was absent in all cases and therefore was excluded from further analysis. Interestingly, ATP6V1G1 expression was also high in commercially available GBM cells (LN229 and T98G) compared to a less aggressive line (SW1088; Supplementary Figure 3A, 3B), and in high-grade human glioma tissues compared to grade II tumors (Figure 1C).

**Table 1:** Demographic and clinicopathological characteristics of the glioma patients' series

	Grade	Tumor type [abbreviation]	n	Age ^1^ [years]	Gender [M/F]	MGMT methylated cases [n, %]	IDH1^R132H^ [n, %]
Gene expression series [n=54]	Grade II [n=10]	Astrocytoma [Astr]	8	43 [29-65]	6M/2F	5 [62%]	2 [25%]
		Oligodendroglioma [OD]	2	25 [18-31]	1M/1F	1 [50%]	-
	Grade III [n=11]	Anaplastic Astrocytoma [AA]	8	54 [29-78]	5M/3F	3 [37.5%]	1 [12.5%]
		Anaplastic Oligodendroglioma [AO]	2	33 [27-39]	1M/1F	1 [50%]	1 [50%]
		Anaplastic Oligoastrocytoma [AOA]	1	49	1M	1	-
	Grade IV	Glioblastoma [GBM]	33	62 [31-82]	20M/13F	16 [48.5%]	-
Tissue microarray series [n=187]	Grade II [n=60]	Astrocytoma [Astr]	13	41 [19-73]	9M/4F	7 [53.8%]	9 [69%]
		Oligodendroglioma [OD]	47	45 [25-72]	28M/19F	39 [83%]	31 [66%]
	Grade III [n=29]	Anaplastic Astrocytoma [AA]	19	46 [22-78]	11M/8F	10 [52.6%]	8 [42%]
		Anaplastic Oligodendroglioma [AO]	10	52 [34-72]	4M/6F	9 [90%]	8 [80%]
	Grade IV	Glioblastoma [GBM]	98	55 [24-78]	59M/39F	48 [48.9%]	11 [11%]

1 Median patients' age at diagnosis with range is provided

Next, to identify whether V-ATPase subunit expression could be elevated in cancer stem cell enriched samples, we analyzed seven primary neurospheres obtained from GBM patients (Supplementary Table 1) for whom the corresponding tumor tissue and the differentiated primary cell cultures were available (Figure 1D). Elevated gene and protein expression of ATP6V1G1 subunit in neurospheres was detected (Figure 1E, 1F and Supplementary Figure 4A). Moreover, ATP6V1G1 gene and protein expression significantly decreased when GBM neurospheres were differentiated into adherent cell monolayers (Figure 1G-1I and Supplementary Figure 4B-4D). Finally, V1G1 expression was undetectable in protein extract of grade I brain tumors (Figure 1H, 1I and Supplementary Table 1), whereas it was abundant in rat embryonic hippocampal neuronal cells (E18 RHN; Figure 1H, 1I). These data indicate that V-ATPase G1 expression is high in aggressive gliomas and in cancer cell stem cells.

### V-ATPase G1 is a marker of poor prognosis for GBM

**Figure 2:**
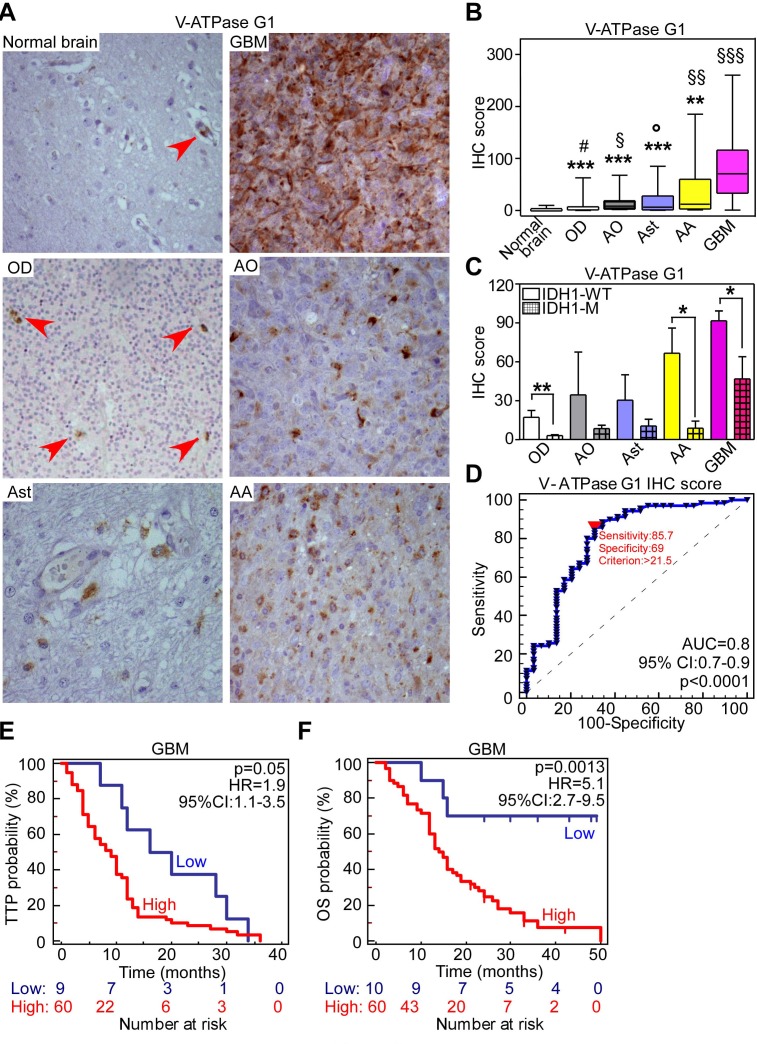
V-ATPase subunit G1 is a novel marker of poor prognosis in GBM patients **A.**, **B.** A glioma tissue microarray comprising 85 cores of normal brain parenchyma was analyzed for V-ATPase G1 expression by immunohistochemistry (IHC) **A.** Photomicrographs representing V-ATPase G1 immunoreactivity in normal brain tissue or in the indicated glioma histotype. Arrowheads indicate brain endothelium. **B.** V-ATPase G1 IHC score was obtained by multiplying the percentage of positive cells (range 0-100%) and the staining intensity (range: 0-3). According to this score, normal brain was almost negative (median IHC score 0; range: 0-10). Oligodendrogliomas (OD; median IHC score 1.75; range: 0-62.5), anaplastic OD (AO; median IHC score 6.1; range: 1.5-67.5), astrocytomas (Astr; median IHC score 6.2; range: 0.25-85), anaplastic astrocytomas (AA; median IHC score 11.75; range: 1-185) displayed higher V-ATPase G1 expression compared to normal brain. ^#^, *p* = 0.0004; ^§^, *p* = 0.047; °, *p* = 0.038; ^§§^, *p* = 0.005; ^§§§^, *p* < 0.0001 by unpaired *t*-test with Welch's correction, respectively. GBMs scored the highest expression for the subunit G1 (median IHC score 70.25; range: 0.5-260) compared to normal brain (§§§, *p* < 0.0001) and to lower grades gliomas (**, *p* = 0.012; ***, *p* < 0.001 by unpaired *t*-test with Welch's correction). **C.** V-ATPase G1 expression according to R132H mutation of IDH1 gene in different glioma histotypes. Bars, mean±SEM. *, *p* = 0.01; **, *p* = 0.009 by unpaired *t*-test. IDH1-WT or M, absence or presence of R132H mutation; **D.** ROC curve was used to identify a cut-off of V-ATPase subunit G1 IHC score (indicated by a red triangle) for patients' categorization into low- or high-expressors. AUC, Area under curve. 95% CI, 95% Confidence Interval. **E.**, **F.** Time to progression (TTP, **E**) or overall survival (OS, **F**) end-points were evaluated in GBM patients according to V-ATPase G1 level using the Kaplan-Meier method. *P* values are from Log-rank test. HR, Hazard Ratio; CI, Confidence Interval.

We next evaluated whether V-ATPase G1 was significantly enriched in GBM and if was correlated to GBM patients' clinical features or prognosis. Therefore we analyzed V-ATPase G1 immunoreactivity in a larger set of primary gliomas arranged in tissue microarrays (TMAs) derived from 187 patients and in 85 normal brain counterparts (Tissue microarray series; Table 1). Generally, V-ATPase G1 was significantly more abundant in tumor tissues compared to normal cerebral parenchyma (Figure 2A, 2B) and GBM displayed the highest immunoreactivity compared to oligodendroglioma (OD; *p* < 0.0001), anaplastic OD (AO; *p* < 0.0001), astrocytomas (Ast; *p* < 0.0001) or anaplastic astrocytomas (AA; *p* = 0.01) (Figure 2B). In normal brain or low-grade oligodendroglioma samples, V-ATPase G1 was expressed by endothelial cells (arrowheads in Figure 2A). Conversely, in grade III gliomas or GBMs the pump subunit was predominantly localized in tumor cells (Figure 2A). In regards to clinical features of gliomas (Table 1), V-ATPase G1 was more expressed by tumors wild-type for IDH1 enzyme (Figure 2C) whereas it did not correlate to MGMT promoter methylation (Supplementary Figure 5). To investigate whether V-ATPase G1 expression could be used as a prognostic marker, we established a cut-off to sort patients into low- and high-V-ATPase G1 expressing categories using ROC curves (Figure 2D and Supplementary Figure 6). According to this cut-off, corresponding to an immunohistochemistry score of 21.5, GBM patients with high V-ATPase G1 expression had the worst outcome (Figure 2E, 2F and Supplementary Table 2). More importantly, multivariate analysis identified V-ATPase G1 expression as a predictor of shorter overall survival for GBM patients, independent of clinical or molecular features of disease (*p* = 0.005 by Cox regression analysis; Table 2).

**Table 2:** Multivariate analysis Overall survival (OS) was analyzed in GBM patients according to the indicated covariate^2^ using the Cox Proportional-Hazards regression model

Analysis	Covariate^3^	*P*	HR^4^	95% CI^5^
OS [n=70]	Age at diagnosis [≤50y]	0.007	0.5	0.2–0.8
	MGMT methylation	0.07	1.7	1–3
	KPS ^6^	0.09	0.9	0.9–1
	IDH1^R132H^	0.1	0.2	0.03–1.9
	V-ATPase G1 score	0.005	3.5	1.1–11.4

2 Only covariates significant at univariate level were considered (see Supplementary Table 2 for details)

3 All covariates were categorical. For patients' age, 50 years of age was used as cut-off; for V-ATPaseG1 patients were grouped in low or high expressors according to the protein level established by ROC analysis.

4 HR, Hazard Ratio

5 CI, Confidence interval

6 KPS, Karnofsky Performance Score

### ATP6V1G1 depletion or V-ATPase inhibition selectively decrease viability of primary GBM neurospheres

**Figure 3:**
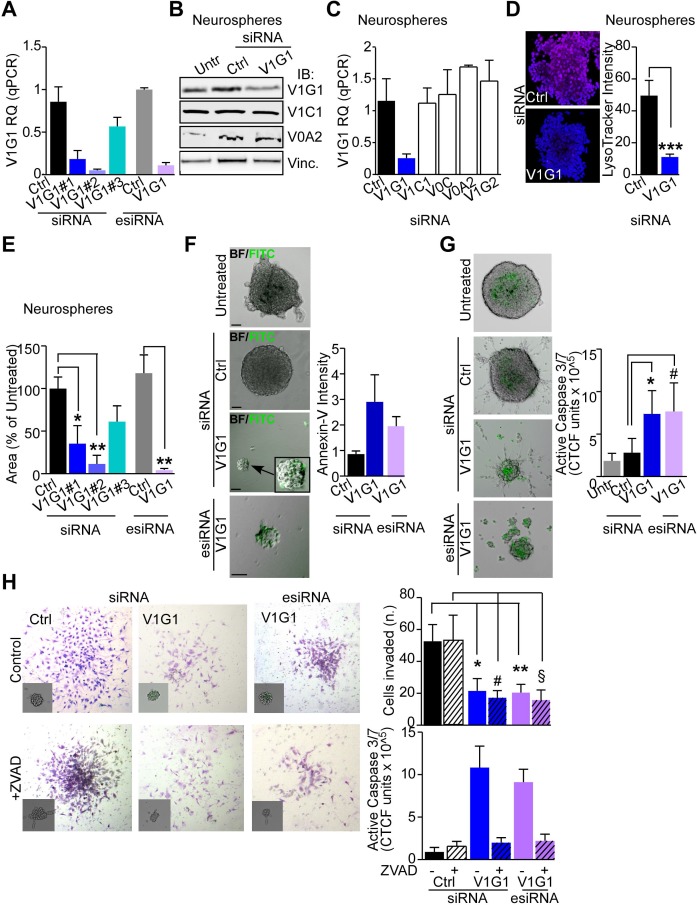
V-ATPase subunit G1 is central for GBM stem cell viability and invasive potential **A.** Optimization of ATP6V1G1 transient knockdown in neurospheres by small interfering RNA (siRNA) was performed with three different targeting siRNAs or with esiRNA technology. The small interference RNA #1 (V1G1#1) was then chose for subsequent analyses together with V1G1-directed esiRNA unless otherwise indicated. **B.** Immunoblotting analysis of the indicated V-ATPase subunits in GBM neurospheres left untreated (Untr) or silenced with a control (Ctrl) or an ATP6V1G1-directed siRNA. A representative experiment is shown. **C.** ATP6V1G1 gene expression quantification in GBM neurospheres transfected with the indicated siRNA (*n* = 7). **D.** Confocal immunofluorescence images of live GBM neurospheres transfected with a non-targeting (Ctrl) or an ATP6V1G1-directed siRNA (V1G1) for 48 hours and then incubated with LysoTracker Red^590^ for 10 minutes at 37°C. Maximum intensity projections are shown for a single representative experiment out of six repeats. Original magnification x 400. *Right*, quantification of LysoTracker mean fluorescence intensity. **, *p* = 0.01 Mann-Whitney U test. **E.** GBM neurospheres were transfected with controls or targeting V1G1 siRNAs or esiRNA and their area was calculated 48 hours later. Neurospheres area is expressed relative to untreated cultures. **F**, **G.** Apoptosis induction or activation of caspase 3/7 after ATP6V1G1 knockdown (V1G1 siRNA or esiRNA) was evaluated by Annexin V staining or active caspase 3/7 probe and fluorescence microscopy, respectively. *Right,* Quantification of mean fluorescence intensities (*n* = 6). Scale bar indicates 100μm. *, *p* = 0.016; #, *p* = 0.02 by Mann-Whitney U test. **H.** Neurospheres silenced for ATP6V1G1 or with a scramble molecule (Ctrl) and pre-incubated with the general caspase inhibitor Z-VAD-FMK (ZVAD). or its negative control Z-FA-FMK (Control) were subjected to matrigel invasion for 24 hours after which invaded cells were stained with toluidine blue and counted. Representative images of an experiment out of six are shown. *Insets*, caspase activation was monitored by CellEvent Caspase 3/7 probe (green) by fluorescence microscopy and quantified as corrected total cell fluorescence (CTCF). Data are expressed as percentage of invaded spheres relative to the input. *, *p* = 0.02; #, *p* = 0.03; **, *p* = 0.004 ;§, *p* = 0.01 by Mann-Whitney U test.

Prompted by the up-regulation of V-ATPase G1 in GBM, we next asked whether knock-down affected proton pump function and cells viability. To address this, we used different GBM models, including LN229 GBM cells, primary GBM monolayer cultures differentiated from neurospheres or patients' derived neurospheres (Supplementary Figure 4). Transient ATP6V1G1 silencing by siRNAs or esiRNA (Figure 3A and Supplementary Figure 7A) effectively decreased protein (Figure 3B) and mRNA levels while expression was not affected by the knock-down of other V-ATPase subunits (Figure 3C and Supplementary Figure 7B). Interestingly, ATP6V1G1 silencing was sufficient to impair V-ATPase-mediated acidification of lysosomes in GBM neurospheres (Figure 3D). In contrast, in differentiated GBM monolayer or LN229 cultures ATP6V1G1 knock-down marginally reduced lysosomal pH and lysosomal degradation (Supplementary Figure 7C, 7D). Depletion of ATP6V1G1 decreased average neurosphere areas (Figure 3E), induced apoptosis (Figure 3F and Supplementary Figure 8A), and activated effector caspase 3 and 7 (Figure 3G and Supplementary Figure 8B). Cell death triggered by V1G1 depletion did not involve the Bcl2 pro-apoptotic member Bax and did not significantly alter the cell cycle (Supplementary Figure 8C, 8D, respectively). Importantly, V1G1 depletion decreased neurosphere invasion through matrigel (Figure 3H) independently of caspases activity (Figure 3H, +ZVAD samples).

**Figure 4:**
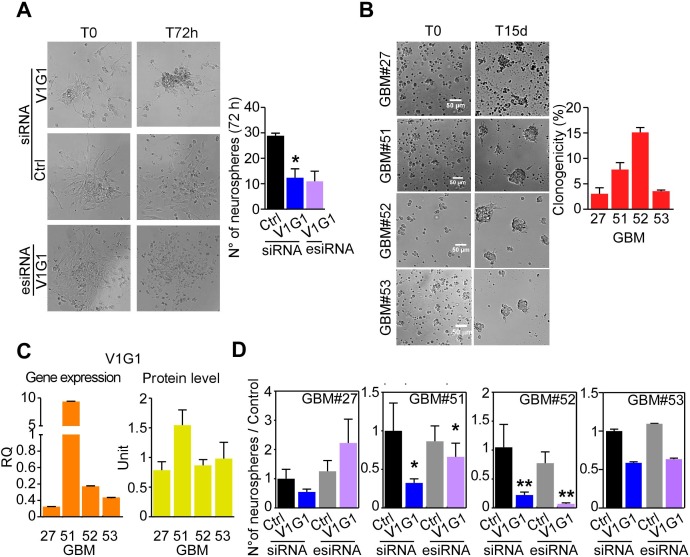
V-ATPase G1 levels are associated to clonogenicity of GBM neurospheres **A.** The ability of GBM cultures to generate neurospheres after specific ATP6V1G1 knockdown (V1G1-siRNA or esiRNA) relative to control sample was evaluated by time lapse microscopy for 72 hours. Representative images captured from movies (Supplementary Movies 1-3) at time zero and at harvesting are shown. **B.** GBM neurospheres clonogenic capacity in methylcellulose containing media was analyzed in the indicated samples. *Left*, quantification of spheres containing at least 20 cells. **C.** V-ATPase G1 gene or protein expression levels in the indicated GBM cultures. **D.** The clonogenic potential of the indicated GBM neurosphere was assessed after V1G1 knockdown by siRNA or esiRNA and compared to corresponding controls. *, *p* < 0.05; **, *p* < 0.01 by Mann Whitney U test. For all graphs, bars represent mean ±SEM of at least six experiments.

In contrast, in adherent glioma cultures or LN229 cells, cell death, caspase 3/7 activity or cell cycle transition was not affected by ATP6V1G1 levels (Supplementary Figure 9A-9F). Cell motility of LN229 cells after ATP6V1G1 knock-down was modestly impaired (Supplementary Figure 9G, 9H), with a reduction of invaded cells of about 30% (Supplementary Figure 9H), whereas cell invasion was not impaired by V1G1 depletion in differentiated GBM cultures (Supplementary Figure 9I). Finally, we monitored sphere-forming ability of primary GBM cells transfected with a control, an ATP6V1G1-directed siRNA or esiRNA over a period of 72 hours. ATP6V1G1 depletion severely restrained formation of neurospheres, compared to control transfected or untreated samples (Figure 4A and Supplementary Movies). As expected [18], GBM neurospheres from different patients displayed heterogeneous clonogenic potential (Figure 4B). We therefore analyzed whether V-ATPase G1 levels were correlated with spheres clonogenicity also in response to ATP6V1G1 gene knockdown. Remarkably, V1G1 was more enriched in GMB patients with higher sphere-forming ability (Figure 4B, 4C), and a lower ATP6V1G1 gene expression level (GBM#27 and 53 samples) was associated to reduced effect of V1G1 knockdown on sphere-forming ability of GBM cultures (Figure 4D).

**Figure 5:**
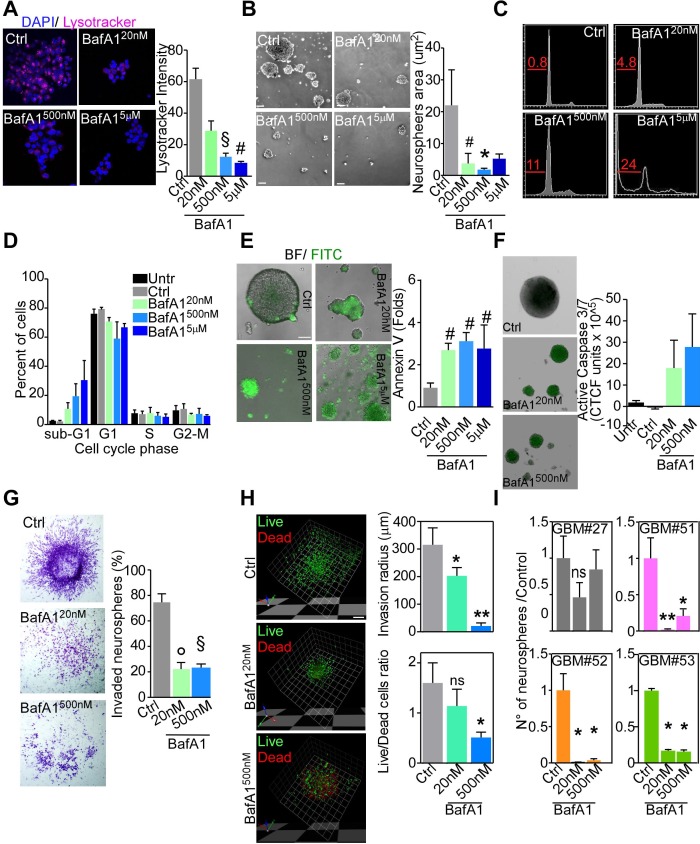
V-ATPase targeting through bafilomycin A1 abrogates GBM stem cells **A.** Confocal immunofluorescence images of live GBM neurospheres treated with different concentration of bafilomycin A1 (BafA1) for 48 hours and then incubated with LysoTracker Red^590^ for 10 minutes at 37°C. Maximum intensity projections are shown for a single representative experiment out of six repeats. Original magnification x 400. *Right*, quantification of mean fluorescence intensity. ^§^, *p* = 0.0004; ^#^, *p* = 0.0001. **B.**-**F.** GBM neurospheres treated with different doses of BafA1 for 48 hours were analyzed for dimensions **B.**, DNA content by propidium iodide staining and flow cytometry **C.**, **D.** induction of programmed cell death (Annexin V staining; **E.**), activation of caspase 3/7 by fluorescent microscopy **F.**, or cell invasion through matrigel by direct cell counting **G.**. *, *p* = 0.016; ^#^, *p* = 0.03; °, *p* = 0.013; ^§^, *p* = 0.027. For all graphs, bars represent mean ±SEM of at least six experiments. **H.** GBM neurospheres were embedded in collagen and treated as indicated. After 48 h cultures were stained with calcein-AM (live cells, green) and ToPro-3 (dead cells, red) and analyzed by confocal microscopy. *Right*, Quantification of the invasion radius and of the live *vs* dead cells ratio. Bars, mean±SEM (*n* = 4). *, *p* = 0.02; **, *p* = 0.004. **I.** GBM neurospheres clonogenicity after BafA1 treatment was assessed by methylcellulose assay for the indicated cultures. Bars, mean ±SEM (*n* = 3). All statistics are from Mann-Whitney U test.

Spurred by the effects of V-ATPase G1 knock-down on GBM samples, we explored inhibition of V-ATPase activity with the selective inhibitor bafilomycin A1 (BafA1), as a strategy to hamper GBM cell viability. Acute treatment (48 hours) of GBM neurospheres or monolayers with different concentrations of BafA1 (20nM-5μM range) strongly reduced lysosomal acidification (Figure 5A, Supplementary Figure 10A, 10B). This effect was accompanied by a significant reduction of neurospheres area (Figure 5B). Quantification of DNA content in neurospheres treated with BafA1 revealed that toxicity progressively increased with the drug concentration, with approximately 25% of the cells in sub-G1 phase upon acute treatment with 5μM BafA1 (Figure 5C, 5D). As determined by annexin V staining, apoptosis was activated even upon treatment with low dose of BafA1 (Figure 5E). Altogether, these data suggest that at high BafA1 doses cell death might occur through both apoptotic and necrotic mechanisms. Therefore, to avoid unspecific cell death, we used 20nM or 500nM of BafA1 for subsequent experiments. Confirming annexin V data and similar to what observed for ATP6V1G1 knockdown, BafA1 induced activation of Caspase 3/7 at both concentrations (Figure 5F). Besides inducing programmed cell death, BafA1 strongly inhibited motility of GBM neurospheres (Figure 5G). Indeed, calcein AM/ToPro-3 differential staining of collagen-invading cells indicated that at 20nM migration is impaired in presence of very low level of dead cells, suggesting that V-ATPase inhibition might diminish GBM stem cells motility independently from cell death (Figure 5H).

Finally, impairment of clonogenic potential by BafA1 treatment inversely reflected V-ATPase G1 levels detected in patients' derived GMB neurospheres (Figure 5I; see Figure 4C for V1G1 levels). Conversely and strikingly, neither viability of differentiated GBM cell cultures (Supplementary Figure 10C-10E) or of commercial LN229 and T98G cells (Supplementary Figure 10F, 10G), nor motility of differentiated GBM cultures (Supplementary Figure 10H) was affected by acute treatment with BafA1 at any dose. Overall these data indicate that V-ATPase pump function is required specifically by GBM neurospheres to preserve their tumorigenic potential and their propagating ability.

### V-ATPase inhibition decrease viability of GBM organotypic cultures

**Figure 6:**
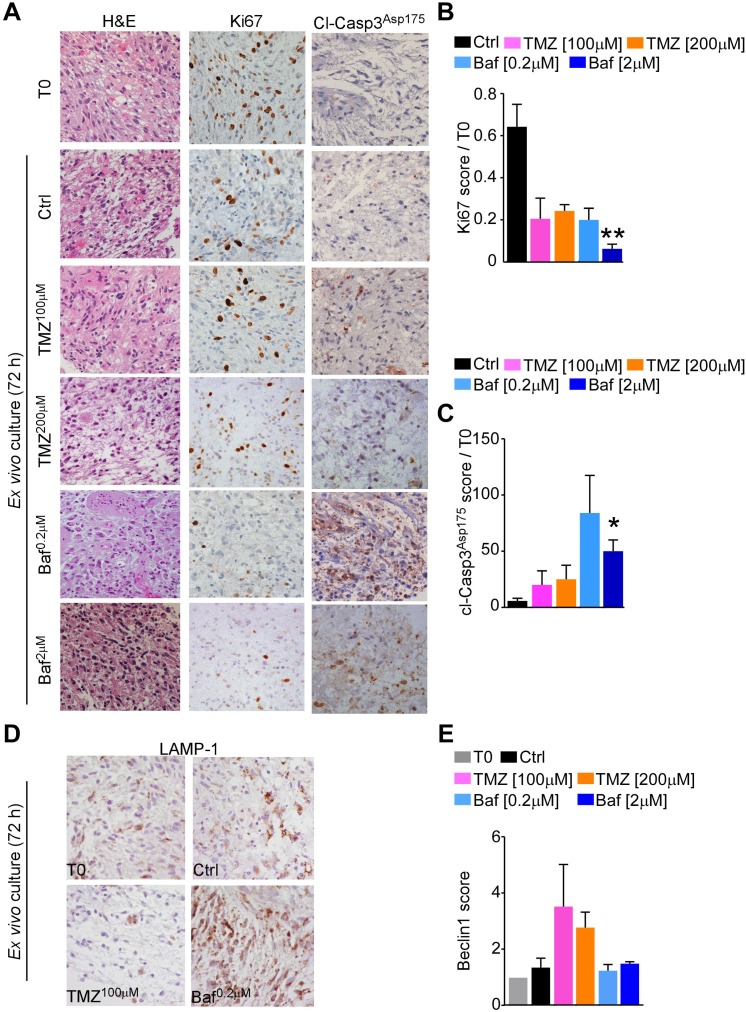
Bafilomycin A1 treatment decreases cell viability in GBM tissue cultures Organotypic tissue cultures from eight GBMs were treated up to 72 h with different concentration of temozolomide (TMZ) or Bafilomycin A1 (Baf) and tissue proliferation (Ki67 labeling, **A.**, **B.**), or apoptosis (Cleaved Caspase-3, **A.**, **C.**) was scored in each sample relative to uncultured (T0) sample. *, *p* = 0.03; **, *p* = 0.007 by Mann-Whitney U test. H&E, Hematoxylin and Eosin. **D.**, **E.** GBM tissue cultures treated as in A were analyzed for lysosomal content (LAMP-1, **D.**) or for expression of the autophagy-related protein Beclin 1 (**E.**) by immunohistochemistry. Photomicrographs show representative GBM cultures. Original magnification x400. Data are presented as mean ±SEM.

To test the feasibility of V-ATPase inhibition in experimental set-up closest to the *in vivo* GBM situation, we used patient-derived organotypic glioma cultures which preserve the tri-dimensional tissue architecture and cellular heterogeneity of the original tumor [24, 25]. We therefore performed BafA1 treatment in a set of eight glioblastoma organotypic cultures (Supplementary Table 3), to compare drug effects on glioma viability and proliferation with the alkylating agent temozolomide (TMZ), which is the standard of care for GBM. After 72 hours, treatment with BafA1 significantly decreased glioma proliferation (Ki67 labelling; *p* = 0.02; Figure 6A, 6B) and induced apoptosis (cleaved Caspase-3^Asp175^ expression; *p* = 0.03; Figure 6A, 6C) while TMZ had little effect (Figure 6A-6C).

BafA1 and TMZ are known to interfere with autophagy, with TMZ inducing autophagosome formation and BafA1 blocking autophagosome fusion with lysosomes [26]. As previously documented in neuroblastoma cells in culture [27], treatment of GBM organotypic cultures with BafA1 increased lysosome-associated membrane protein-1 (LAMP-1) content (Figure 6D), indicating induction of lysosomal stress. However, levels of beclin1, a regulator of autophagosome formation, in GBM organotypic cultures treated with BafA1 did not change, suggesting that lysosomal stress did not involve modulation of tumor autophagy (Figure 6E). Therefore, our data do not support the possibility that autophagy is a mediator of cell death in GBM organotypic cultures treated with BafA1. Overall, these data indicate that V-ATPase inhibition is more efficient than TMZ treatment to reduce viability of GBM tissue.

### Treatment of GBM neurospheres or organotypic cultures with BafA1 induces selective stem cell depletion and reduces stem cell factors expression

**Figure 7:**
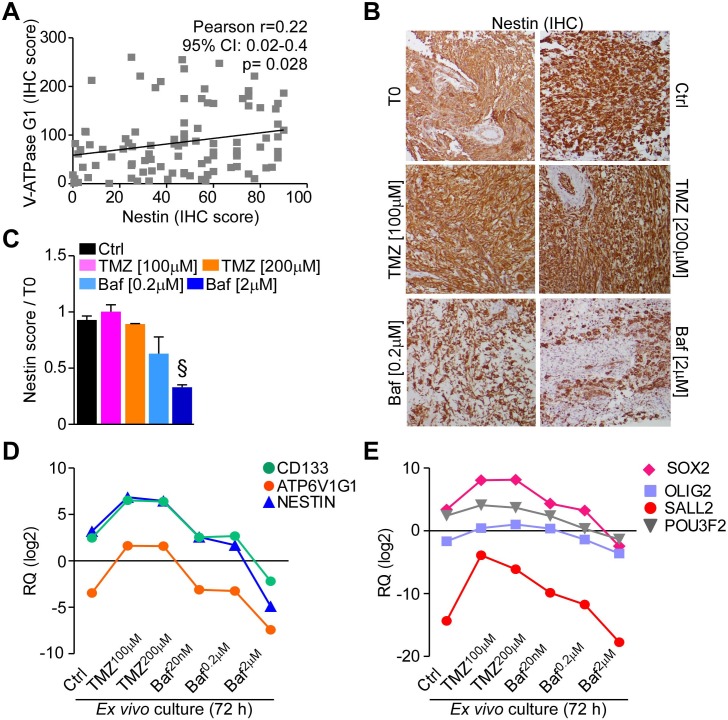
Bafilomycin A1 reduces stem cell factors expression in GBM organ cultures **A.** Nestin immunoreactivity was analyzed in GBMs of the tissue micro-arrays and correlated to V-ATPase G1 expression in the same samples. **B.**, **C.** Short term GBM organ cultures were treated as in Figure 6. Nestin presence was assessed after 72 hours and quantified relative to uncultured samples (T0). Original magnification x400. Bars, mean±SEM. §, *p* = 0.018 by Mann-Whitney U test. **D.**, **E.** The stem cell markers CD133, NESTIN or ATP6V1G1 (**D.**) and the neurodevelopmental transcription factors SOX2, OLIG2, SALL2 and POU3F2 (**E.**) were analyzed at gene expression level by qPCR in GBM organ cultures treated as indicated. Each point represents the mean of three cultures. RQ, mRNA Relative Quantity.

**Figure 8:**
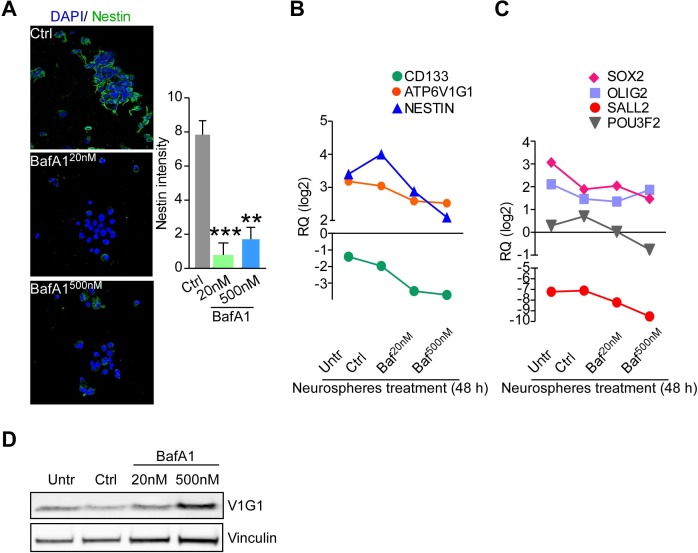
Bafilomycin A1 repress stemness features of GBM neurospheres **A.** Nestin expression was evaluated by confocal immunofluorescence in GBM neurospheres incubated for 48 hours with vehicle or different concentrations of bafilomycin A1 and quantified in multiple z-stacks by ROI. **, *p* = 0.007; ***, *p* = 0.0006 by Mann-Whitney U test. Bars, mean ±SEM (*n* = 5). *Left*, Representative maximum intensity projection images of neurospheres stained for the indicated antibody. **B.**, **C.** Gene expression evaluation of ATP6V1G1, CD133 and NESTIN, or of transcription factors involved in neurodevelopment and reprogramming (SOX2, OLIG2, SALL2 and POU3F2) in GBM neurospheres treated for 48 hours with 20 nM or 500 nM of bafilomycin A1, vehicle (Ctrl), or left untreated (Untr). Each point represents the mean of four experiments. RQ, mRNA Relative Quantity. **D.** V-ATPase G1 protein levels in GBM neurospheres after bafilomycin A1 treatment were analyzed by western blot. Vinculin was a loading control.

Given the results obtained using BafA1 in GBM neurospheres and organotypic cultures, we finally assessed whether BafA1 treatment affected stem cells in GBM tissue. Using the glioma TMA platform (Table 1) we found that V-ATPase subunit G1 expression was significantly correlated with nestin in GBM (*n* = 98, *p* = 0.028; Figure 7A). In addition, treatment with BafA1, but not with TMZ, strongly reduced the number of nestin-positive cells in GBM organotypic cultures (Figure 7B, 7C). These data were confirmed at the gene expression level. Indeed, GBM organotypic cultures treated with the V-ATPase inhibitor displayed decreased levels of NESTIN and CD133 besides showing lesser expression of ATP6V1G1 (Figure 7D). We also analyzed a panel of transcription factors recently associated to GBM stem cell propagation, such as SOX2, OLIG2, SALL2 and POU3F2 [28]. While TMZ increased their expression in treated cultures, variable down-regulation of SOX2, SALL2 and POU3F2 was observed in GBM organotypic cultures incubated with 2μM of BafA1 (Figure 7E). Consistent with the effects observed in organotypic cultures, treatment of GBM neurospheres, which are known to be enriched in cancer stem cells [28], with BafA1 decreased nestin protein (Figure 8A) and mRNA levels (Figure 8B), as well as expression of stem cell-associated factors CD133, SOX2, SALL2 and POU3F2 to different degrees (Figure 8C, 8D), while V-ATPase G1 mRNA and protein levels remained substantially unchanged (Figure 8D). These data confirm that GBM stem cells are highly sensitive to V-ATPase inhibition.

## DISCUSSION

In this study we show that V-ATPase G1 is a novel marker of poor prognosis for glioblastoma patients. Notably, V-ATPase G1 levels were predictive of overall survival in GBM patients independently of the epigenetic silencing of MGMT gene, IDH1 mutation, Karnofsky performance score, or patients' age at diagnosis. Our data reveal that ATP6V1G1 is selectively upregulated in GBM stem cells and that targeting of V-ATPase through specific gene knock-down or pharmacological inhibition hampers GBM viability, tumorigenicity and stem cell factors expression. We also demonstrate that V-ATPase inhibition by BafA1 is feasible and highly effective in human GBM organ cultures, an experimental platform that closely mimics the *in vivo* cancer situation [24, 25]. Given the selective exploitation of V-ATPase by glioma stem cells, our preliminary evidence suggests that V-ATPase targeting could represent an attractive novel therapeutic strategy in GBM, a fatal disease with no curative option. Indeed the suitability of targeting V-ATPase to eradicate cancer stem cells has been previously explored in embryonal rhabdomyosarcoma disease [22].

Overall, we found that V-ATPase G1 protein is progressively overexpressed by high-grade gliomas compared to less aggressive grade II tumors, whereas it is expressed at low level in normal brain. In our series, V-ATPase G1 was not associated to MGMT epigenetic status, but increased protein levels were detected in brain tumors wild-type for isocitrate dehydrogenase 1 (IDH1) enzyme, a known marker of worse prognosis [19], and this occurred irrespective of their grade. The oncogenic role of IDH1 mutations in gliomas is unclear. Nevertheless, IDH enzymes are involved in tumor metabolism and energy (NADP+) production [29]. Our findings may suggest that a regulatory loop between an acidic microenvironment and altered bioenergetics in tumor cells could contribute to tumorigenesis. Further work will be needed to understand the mechanism behind the correlation between V-ATPase and IDH enzymes in glioma cells.

Little is known about regulation of V-ATPase subunits expression during tumorigenesis. A previous report indicates that V-ATPase G1 is expressed ubiquitously in mouse brain, while its paralog V-ATPase subunit G2 is expressed in neurons of the central nervous system where it is specifically involved in acidification of synaptic vesicles [30]. V-ATPase upregulation has been described to induce cell transformation [10], to sustain the cancer stem cell niche [22], and as a defensive mechanism adopted by cancer cells to escape apoptosis induced by anticancer agents [31]. Specifically, V-ATPase activity appears to be required by ovarian or breast cancer cells to invade and metastasize [15, 23, 32-34], or to activate oncogenic signaling [5, 35]. Finally, ATP6V0A encoding a specific subunit associated to V-ATPase function at the plasma membrane, was found upregulated in grade III gliomas [12]. Despite this, the functional relevance of the upregulation of V-ATPase subunits in human cancer is unclear. However, we showed that pharmacologic inhibition of V-ATPase function impact tumor viability and GBM stem cell features, suggesting a clear requirement for pump activity. In good agreement, a recent investigation reported that targeting V-ATPase pump activity hampers growth and tumorigenic potential of rhabdomyosarcoma CSC [22]. In such system, high V-ATPase activity was associated to increased invasive ability and reduced sensitivity to chemotherapeutics, as previously reported [8, 14, 23]. Interestingly, similar to V-ATPase the pH-dependent CLIC-1 channel [36] is required for proliferation of GBM stem cells [18, 21], suggesting that pH unbalances might contribute to GBM stem cell niche maintenance. Altogether, this evidence indicates that V-ATPase might control multiple aspects of tumor development by mechanisms that await elucidation.

In human brain V-ATPase G1 immunostaining strongly resembled nestin pattern in either human glioma or normal tissues. In normal brain or grade II gliomas, both V-ATPase G1 and nestin localize to endothelial cells of blood vessels [37, 38]. Conversely, high-grade gliomas displayed V-ATPase G1 and nestin upregulation in tumor cells, and expression of both proteins was decreased by BafA1. These preliminary data suggest that V-ATPase might be involved in acquisition and/or maintenance of the GBM cancer stem cell niche. Consistent with this, BafA1 has been shown to sensitize glioma stem cells to gamma-radiation reducing their viability and ability to form neurospheres [39].

Concluding, our results suggest that high levels of ATP6V1G1 are a characteristic of GBM stem cells and could influence the onset of chemoresistance typical of high grades gliomas. Indeed patients with a low V-ATPase subunit G1 expression had a longer OS and a better response to adjuvant concomitant chemo-radiation therapy in terms of time to disease progression. Despite increased understanding of the molecular alterations that characterize different subsets of high-grade glioma, almost all patients face a fatal disease with inadequate treatment options. Therefore, there is a crucial need to identify proteins that could be effectively targeted [18]. In this scenario, we have shed light on a core aspect of the cellular machinery that is selectively exploited by GBM cancer stem cells, whose activity can be modulated pharmacologically. Considering that V-ATPase directed drugs are currently under preclinical development [14, 15], our work constitutes the groundwork for future understanding of the antitumor activity of V-ATPase inhibition in gliomas.

## MATERIALS AND METHODS

### Patients

For this investigation, two series of patients diagnosed with *de novo* glioma were enrolled at Fondazione IRCCS Ca' Granda Ospedale Maggiore Policlinico (Milan, Italy). A first series consisted of 54 patients that underwent surgery between 2013 and 2014, of which both fresh and archival neoplastic tissue could be retrieved for molecular or histological analyses respectively (Gene expression series). A second set of 187 patients that consecutively underwent surgery between 2008 and 2010 was enrolled for retrospective investigations on formalin-fixed paraffin-embedded tissues (Tissue microarray series) as previously described [40]. All patients were treated with surgical resection of the lesion and nobody received a merely diagnostic procedure. The Institutional Review Board approved the study. Gliomas were staged according to the WHO classification [41] and clinicopathological or molecular characteristics of both patients' series are described in Table 1. Following histological diagnosis, all patients affected by high grade gliomas (HGG; grades III and GBM) underwent concomitant chemo-radiation therapy according to the Stupp's protocol [42]. Follow-up data was available for GBM patients and it consisted of neuro-oncological assessment and brain MRI with gadolinium every 3 months as described [40]. Tumor progression was defined according to MacDonald's criteria [17]. Time to progression (TTP) and overall survival (OS) records were available for 69 (70%) and 70 (71%) GBM patients, respectively. TTP was calculated from surgery to tumor progression determined by radiological (brain MRIs) procedures, whereas OS was calculated from surgery to patients' death. Follow-up ended in December 2012.

Tissue microarrays (TMAs) of glioma or normal brain tissues were created as previously described [43], with slight modifications. Detailed procedures for TMAs construction and immunohistochemical (IHC) experiments are detailed in the Supplementary Information.

### Oncomine analysis

Differential V-ATPase subunits expression was analyzed in published microarray data sets using a Web-based data-mining platform (Oncomine; available at (http://www.oncomine.org/). Relative expression data of V-ATPase subunits were collected from brain cancer datasets only. The *t*-statistic for significant comparison is provided within the data sets in Oncomine.

### Glioblastoma primary cultures and neurospheres

GBM neurospheres or differentiated monolayer cultures (Supplementary Figure 4B-4D) were obtained as described [18, 44] from seven chemo- and radio-naïve patients surgically treated at Neurosurgery Division (Fondazione IRCCS Ca' Granda Ospedale Maggiore Policlinico) and histologically diagnosed with glioblastoma (WHO grade IV, GBM). Differentiated primary cultures were also derived from two patients diagnosed with grade I glioma (Supplementary Table 1). Post-surgical samples were used after patients' informed consent and Institutional Ethical Committee approval and primary cultures were coded as Grade I#1, 2 and GBM with case number (Supplementary Table 1). Details of this procedure can be found online in the Supplementary Information.

### *Ex-vivo* glioma tissue cultures

Glioma tissue slices were obtained from surgical waste of eight patients affected by GBM treated at Fondazione IRCCS Ca' Granda Ospedale Maggiore Policlinco (Milan, Italy). Written informed consent was obtained from all patients. Clinicopathological characteristics are described in Supplementary Table 3. Briefly, precise thick (300 μm) tissue sections were generated using a vibratome (VT1200, Leica Microsystems, Milan, Italy) as previously described [24, 25], and cultivated up to 72 hours in presence or absence of 100μM or 200μM temozolomide, 0.2μM or 2μM bafilomycin A1 (both drugs were from Sigma-Aldrich, Milan, Italy). As control sample we used an untreated tissue slice cultivated in complete media supplemented with vehicle (1 μl of DMSO in 1 ml of media). Culturing medium supplemented with drugs or vehicle was replaced every 24 h. At harvesting, tissue cultures were snap-frozen or formalin-fixed and paraffin-embedded (FFPE) for molecular or morphological (hematoxylin and eosin staining) and immunohistochemistry analyses, respectively. For each glioma culture, tissue proliferation (Ki67 labeling), apoptosis (cleaved Caspase-3 ^Asp175^), Beclin 1, LAMP-1, Nestin or V-ATPase G1 (clone D-5; Santa Cruz Biotech, Santa Cruz, CA, USA) protein presence and expression in cultivated tissue slices were evaluated by immunohistochemistry (IHC) and scored as percentage of positive cells out of the total. IHC scores in cultivated tissue slices are expressed relative to baseline uncultured sample (T0). Similarly, selected genes expression (CD133, NESTIN, ATP6V1G1, POU3F2, SALL2, OLIG2, SOX2; Supplementary Table 4) were quantified relative to reference gene (18S rRNA) in cultured or not samples. Detailed immunohistochemistry and gene expression protocols can be found in the Supplementary Information.

### RNA purification and qPCR

Total RNA was purified from glioma tissues or cell cultures using Trizol reagent (Life Technologies Inc.) or Master Pure RNA purification kit (Epicentre Biotechnologies, Illumina; Madison, WI, USA) following manufacturer procedures. Detail of retrotranscription or quantitative PCR reactions are provided in the Supplementary Information.

### Cell lines, siRNA transfections and V-ATPase inhibition

SW1088, T98G or LN229 glioma cell lines were purchased from ATCC (Manassas, VA, USA) and cultured as suggested by the manufacturer. Protein extract from E18 primary rat hippocampal neurons (E18 RHN) were a generous gift from Fabio Grohovaz lab (IRCCS San Raffaele Scientific Institute, Neuroscience Division, Milan, Italy). GBM primary cultures or commercial cell lines were transiently transfected in Optimem media using Lipofectamine 3000 (both from Invitrogen, Life Technologies Inc.) and 100pM of specific or non-targeting control siRNAs or esiRNA (all from Sigma Aldrich, St. Louis, MO, USA) as described [45]. Three ATP6V1G1 siRNAs were initially tested (MISSION siRNA SASI_Hs02_00338522; SASI_Hs01_00227261; SASI_Hs01_00227268) together with ATP6V1G1-targeting esiRNA (MISSION esiRNA EHU156241) and corresponding non-targeting control (MISSION esiRNA EHUEGFP). The siRNA SASI_Hs02_00338522 (siRNA-V1G1#1) and V1G1-esiRNA were then chosen for functional experiments if not otherwise indicated. After five hours, transfection mixture was replaced with standard culturing media. V-ATPase inhibition was achieved treating cultures with different concentrations of bafilomycin A1 (BafA1, range 10nM-5μM; Sigma Aldrich) or concanamycin A (ConcA, range 50-500nM; Sigma Aldrich) for 48 hours. Effect of siRNAs or drugs incubation on V-ATPase pump activity was measured incubating primary or commercial GBM cultures with 1 μM of LysoTracker Red (DND-99, Life Technologies Inc.) for 10 min or with 1 μM of DQ-Red BSA (Life Technologies Inc.) for 1 h, as described [35]. Then cultures were rinsed and mounted immediately (Lysotracker) or fixed with 4% paraformaldehyde (4% PFA; DQ-BSA) for confocal microscopy examination (TCS SP5 Confocal, Leica Microsystems, Milan, Italy).

### Cell viability assays

LN229 or T98G cell viability after V-ATPase inhibition by selective drugs was analyzed after 48 h by measuring mitochondrial function through the reduction of 3-(4,5-dimethylthiazol-2-yl)-2,5-diphenyltetrazolium bromide (MTT, Sigma-Aldrich) as reported [45]. Cell cycle transitions and quantification of hypodiploid DNA content (i.e. sub-G1 fraction) were determined in transfected or drug treated cultures after 48 h, by propidium iodide staining and flow cytometry, as described [45]. Alternatively, cells were stained with Annexin V/PI using the FITC Annexin V Apoptosis Detection Kit (BD Bioscience, San Diego, CA, USA), evaluated by multiparametric flow cytometry and analyzed using FlowJo software. For GBM neurospheres the FITC Annexin V Fluorescence Microscopy Kit (BD Bioscience) was used and signal intensity was acquired using a fluorescence inverted microscopy (DMI4000B, Leica Microsystems). Detection of effectors caspase activation was performed incubating live cultures with the CellEvent Caspase-3/7 Green probe (Life Technologies) and signal (at 503nm) or bright-field images were acquired using a fluorescence microscope. Details of the procedure can be found in the Supplementary Information.

### Cell motility and cell invasion

LN229 cells grown in adhesion were transfected with ATP6V1G1 siRNA or control molecule for 24 h after which 5×104 cells were seeded in serum-free medium on collagen- or Matrigel-coated chambers (BD Biosciences) to evaluate cell migration or invasion, respectively. For GBM neurospheres invasion assay, an average of 13 spheres per condition (untreated, vehicle, bafilomycin A1-treated samples or control- and ATP6V1G1 knockdown samples) was used. For ATP6V1G1 or control-silenced glioma cultures the standard culturing medium was used as chemoattractant in the bottom chamber. When bafilomycin A1 effect was tested, complete Neurocult media (Stemcell Technologies) supplemented with the indicated concentration of the drug was used as chemoattractant. Cells or neurospheres were allowed to migrate or invade for 24 h, as described [45] after which cultures present on lower insert surface were fixed with methanol, stained with toluidine blue, and quantified by direct cell counting using bright-field optical microscopy (Leica DMI4000B). For neuropsheres, the number of invaded foci was normalized on the original input. GBM neurospheres invasion experiment was repeated six times in duplicate using six different GBM cultures, whereas other motility experiments were performed three times in duplicate. When neurospheres were preincubated with the general caspase inhibitor Z-VAD-FMK (ZVAD), or its negative control inhibitor Z-FA-FMK (Control; both from BD Pharmingen), 10μM of the indicated molecule was added to the cultures one hour before the seeding on matrigel inserts. Effective inhibition of active caspase 3/7 was analyzed using CellEvent caspase 3/7 fluorescent probe (Life Technologies) in parallel plates when matrigel inserts were harvested. For analysis of contextual tumor cell viability and motility, neurospheres were embedded in 600 μl of bovine collagen type I (Sigma Aldrich) in 24-well plates, overlaid with 1 ml of growth medium containing 20nM of bafilomycin A1 or vehicle (DMSO, control). After 48 h, neurospheres were analyzed for changes in maximum invasion distance (radius) and quantification of live *versus* dead cells was performed staining the spheroids with calcein-AM (live, green) and ToPro-3 (dead, red; both reagents were from Life Technologies) as described [46]. Samples were imaged with a HCX PL Fluotar 10X/0.0NA objective using a Leica TCS SP5 confocal microscope, and multiple stacks (Z-step = 4μm) were generated in 2 channels. 3-D reconstruction of stained neurospeheres was performed using Volocity 3D Image analysis software (PerkinElmer, Waltham, MA, USA).

### Immunofluorescence and time-lapse microscopy

For immunofluorescence experiments, glioma monolayer cultures were grown on cover-glasses whereas neurospheres were cyto-spinned on charged slides (Thermo Scientific, Waltham, MA, USA). Glioma cultures were stained with primary antibodies against V-ATPase G1 (D5, Santa Cruz Biotechnologies), GFAP (G9269; Sigma Aldrich), cleaved Caspase-3 (D175; Cell Signaling Technologies, Danvers, MA, USA), Nestin (MAB 1259; R&D Systems, Abingdon, UK), Sox2 (D6D9, Cell Signaling Technology Inc.), Oligodendrocyte marker O4 (O7139; Sigma Aldrich), GFAP (G9269; Sigma Aldrich), or Tuji1 (T3952; Sigma Aldrich). Detailed procedures are available in the Supplementary Information. Confocal images were generated with a Leica TCS SP5 Confocal microscope (Leica Microsystems). For each sample, analyses of fluorescence intensities was performed in at least five randomly picked fields per condition using ImageJ software (http://imagej.nih.gov/ij/) and experiments were repeated three or 6 times for monolayers cultures or GBM neurospheres, respectively. For Annexin V or Lysotracker quantification the fluorescence threshold was set, and the mean intensity of the fluorochrome was calculated and plotted in the graph. For proteins staining quantification, the area of the cells or the spheres was defined using a ROI as a boundary. We calculated the integrated intensity of the whole ROI. Detailed procedures can be found in the Supplementary Information section. Time-lapse microscopy experiments were performed using three primary GBM cell monolayers cultivated in stem cell permissive media in 48-wells plates. Briefly, GBM cultures at the second or third culturing passage were transfected with a control- or ATP6V1G1-directed siRNA (Sigma Aldrich) as described above, or left untreated. Then GBM cells were monitored over a period of 72 hours for spheres formation using a time-lapse microscope (Eclipse Ti-E, Nikon Instruments, Florence, Italy). Images for each sample were snapped every two hours and then assembled to a movie in QuickTime format.

### Statistical analysis

Groups' comparisons were performed using unpaired two-sided Student's *t* test (with Welch's correction if groups had unequal variances) or Mann-Whitney U test when appropriate (GraphPad Prism software, La Jolla, CA, USA). The significance of a variable for GBM patients' prognosis was analyzed using the Cox regression hazard model either as univariate or multivariate analysis (MedCalc software, Mariakerke, Belgium) considering proteins IHC scores, MGMT methylation status, age at diagnosis, presence of IDH1^R132H^ mutation or tumor grade as categorical variables. To generate a cut-off for V-ATPase G1 IHC score we used receiver operating characteristics (ROC) curves and the non-arbitrary criterion derived from the Youden index (J, MedCalc Software) as described [40]. Glioma patients were then sorted into low or high-expressor categories. The Kaplan Meier method was used to plot survival curves of glioblastoma patients. In TTP analysis patients' death were censored. Difference in survival curves was computed using the Log Rank test (MedCalc Software).

Two-sided *P* values less than 0.05 were considered statistically significant.

## SUPPLEEMENTARY MATERIAL TABLES, FIGURES AND MOVIES








