# Supplementary material for: The vacuolar H+ ATPase is a novel therapeutic target for glioblastoma
Source: Oncotarget. 2015 May 22;6(19):17514–31. doi: 10.18632/oncotarget.4239 (PMC4627325; doi:10.18632/oncotarget.4239)
Supplement: Supplementary file 1 [file oncotarget-06-17514-s001.pdf]

# The vacuolar H+ ATPase is a novel therapeutic target for glioblastoma

## Suppleementary Material

**Tissue micro arrays (TMAs) construction**. Representative tissue blocks from each patient were used to construct TMA, as previously described [36]. Briefly, four tumor areas were selected from each donor tissue block so that all patients were arranged in five TMAs. When present, a core of non-neoplastic brain parenchyma from each case was included in the TMAs. Globally, 85 cores of normal brain were available. For quality control, a 4-µm-thick section was cut from each TMA block and stained with hematoxylin and eosin (H&E) before being analyzed by immunohistochemistry (IHC).

**Immunohistochemistry (IHC).** Sections (4-µm thick) were cut from each TMA block. For antigen retrieval slides were microwaved for 35 minutes in citrate solution, followed by incubation with primary antibodies specific to human ATP6V0A2 (SAB2701067; Sigma-Aldrich, Milan, Italy), ATP6V0C (SAB2105622; Sigma-Aldrich), ATP6V1C1 (HPA023943; Sigma-Aldrich), ATP6V1G1 (D-5; Santa Cruz Biotechnology, Santa Cruz, CA, USA), Cleaved Caspase-3 (Asp175) (9661, Cell Signaling Technology Inc., Danvers, MA, USA), Ki67 (30-9, CONFIRM Ki-67, Ventana Medical Systems Inc., Roche Group, Tucson, AR, USA), Beclin 1 (G-11, Santa Cruz Biotechnology), LAMP-1 (clone H4A3, BD Biosciences, San Jose, CA), or to human Nestin (MAB1259; R&D Systems; Minneapolis, MN, USA) for 1 hour at 22°C. Negative controls were prepared in the absence of primary antibody and included in each reaction. IHC was performed using an automatic stainer (Benchmark ULTRA, Ventana Medical Systems Inc., Roche Group, Tucson, AR, USA), with detection of antibody reactivity with a kit using peroxidase-diaminobenzidine as the chromogen (DAB UltraView, Ventana). All slides were counterstained with hematoxylin. Immunoreactivity for all antibodies in the various samples was independently evaluated by two investigators (SF and VV) and independently scored for cytoplasmic or nuclear

(Ki67) localization. When discrepancies in scoring occurred, a consensus interpretation was reached after reexamination. For Beclin 1 or LAMP-1 the staining intensity was evaluated as: 0, no staining; 1, weak, 2, moderate; or 3, strong. A two-score system for percentage of positive cells and intensity of staining was used to quantify the reactivity for all V-ATPase subunits and Nestin and multiplied to generate a score (IHC score range 0-300) as described. For Ki67 labeling index, the percentage of positive cells was calculated out of the total number of tumor cell per sample and it was used as a measure of tumor proliferative activity [23].

**Glioblastoma primary cultures and neurospheres.** Briefly, post-surgical GBM samples were enzymatically and mechanically processed using the Tumor Dissociation Kit and the gentleMACS Dissociator (Miltenyi Biotec, San Diego, CA, USA) to reach a single-cell suspension. Then GBM primary cultures were grown either in stem cell permissive Neurocult medium enriched with Human NS-A Proliferation Kit, 10ng/ ml bFGF and 20ng/ml EGF (Stemcell Technologies, Grenoble, France), or in differentiating RPMI media supplemented with 5% FBS (Gibco-Invitrogen, Life Technologies Inc., Carslbad, CA, USA) to obtain neurospheres or monolayers, respectively. Sphere formation occurred within 2 weeks of culture after which their self-renewal capacity, tumorigenicity and markers expression was tested. Briefly, newly formed spheroids were dissociated to single-cell suspension, re-plated in stem cell-permissive conditions and allowed to generate spheres before being employed in experiments. To measure neurospheres clonogenicity, cells were resuspended in basal Neurocult media containing methylcellulose (R&D Systems; Abingdon, UK) and seeded on 35-mm culture plates at a density of 2000 cells/ml. Three plates per condition were used and colonies containing more than 20 cells were scored two weeks after plating as described (Setti JNCI). Clonogenic capacity (percentage of neural stem cells per plate) was recorded as the ratio between neurospheres formed and the initial number of single cells plated in treated samples relative to controls. When differentiation of GBM neurospheres into monolayers was performed, spheres well cultivated with Neurocult medium supplemented with Human NS-A Differentiation Supplement (Stem Cell Technologies Inc.) for 30 days. Neurospheres tumorigenicity

was assessed by matrigel invasion assay (BD Bioscience). Expression of astroglial or stem cell markers was verified by qPCR (not shown) or immunofluorescence analyses (Supplementary Fig. 4E).

**Immunofluorescence**. For active caspases 3/7 detection, live cultures were incubated fro 30 minutes with CellEvent Caspase-3/7 Green Detection Reagent (Life Technologies) and then analyzed by fluorescence microscopy. Analysis was then performed using Image J on selected ROI calculating the Corrected Total Cell Fluorescence (CTCF) with the formula: CTCF= Integrated Density – (Area of selected cell X Mean fluorescence of background readings) as described [47]. For immunostaining, cells were fixed with 4% paraformaldehyde (PFA) in phosphate-buffered saline (PBS). Cells were then permeabilized in 0.1% Triton-X for 10' and blocked with 5% Bovine Serum Albumin (BSA) for 30' followed by incubation with primary antibodies o.n. at 4° degrees. Alexa Fluor 488- or Alexa Fluor 568-conjugated secondary antibodies (Life Technologies Inc.) were applied at RT for 60'. Nuclei were counterstained with Hoechst33342 (1:5000) and slides were mounted using ProLong Gold Antifade reagent (Life Technologies Inc.).

**RNA retrotranscription and qPCR.** After total RNA purification from glioma tissues or cell cultures, 300 ng of total RNA were reverse transcribed using the High Capacity cDNA Reverse Transcription Kit with random hexamers and 8 ng of cDNA per reaction was used for qPCR experiments. 18S ribosomal RNA (18s_rRNA) or TATA-Box Binding Protein (TBP) were use as endogenous control for target genes relative quantification (RQ). A detailed list of assays is shown in Supplementary Table 4. Target genes RQ were calculated using the 2^-DCt formula, normalized on their median expression value and log2 transformed as described [45].

## Supplementary Reference

47. Burgess A, Vigneron S, Brioudes E, Labbé J-C, Lorca T & Castro A (2010) Loss of human Greatwall results in G2 arrest and multiple mitotic defects due to deregulation of the cyclin B-Cdc2/PP2A balance. Proc Natl Acad Sci USA. 2010; 107**:** 12564–69

# Supplementary Tables

**Supplementary Table 1.** Clinicopathological features of GBMs from which primary cell cultures were derived.

| Culture ID | Gender | Age (y) | Ki67 (%) | MGMT[1] | 1p/19q LOH[2] | IDH1[3] | Neurospheres | Differentiated cultures |
|---|---|---|---|---|---|---|---|---|
| Grade I#1[4] | F | 19 | 5% | UM | Neg | - | - | X |
| Grade I#2[4] | M | 18 | 2% | - | - | - | - | X |
| GBM27 | F | 82 | 15 | UM | 1p | WT | X | X |
| GBM32 | M | 42 | 65 | UM | Neg | WT | X | - |
| GBM51 | F | 78 | 35 | M | Neg | WT | X | X |
| GBM52 | M | 59 | 20 | UM | Neg | WT | X | X |
| GBM53 | M | 53 | 15 | UM | Neg | WT | X | X |
| GBM62 | M | 64 | 50 | UM | Neg | WT | X | - |
| GBM63 | M | 55 | 25 | UM | Neg | WT | X | X |

[1] M, Methylated; UM, Unmethylated
[2] Neg, absence of LOH at the indicated loci; np, not performed
[3] WT, Wild Type
[4] Diagnosis of Grade I #1 and #2 glioma was Papillary glioneuronal tumor and Ganglioglioma, respectively

**Supplementary Table 2.** Univariate analysis was performed using the Cox proportional-hazards regression model to assess prognostic potential of the indicated covariate in GBM patients according to time to progression (TTP) or overall survival (OS) endpoints.

| Analysis[1] | Follow-up[2] | Covariate[3] | *P* | HR[4] | 95% CI[5] |
|---|---|---|---|---|---|
| TTP (n=69) | 10 (9-13) | Age at diagnosis (≤50y) | 0.009 | 0.5 | 0.3-0.8 |
| | | MGMT (UM) | 0.06 | 1.6 | 0.9-2.6 |
| | | IDH1$^{R132H}$ | 0.6 | 0.7 | 0.3-2.2 |
| | | KPS[6] | 0.07 | 1 | 0.9-1 |
| | | V-ATPase G1 score (High) | 0.053 | 2 | 0.9-4.2 |
| OS (n=70) | 16 (15-22) | Age at diagnosis (≤50y) | 0.01 | 0.4 | 0.2-0.8 |
| | | MGMT (UM) | 0.012 | 2 | 1.2-3.5 |
| | | IDH1$^{R132H}$ | 0.03 | 0.2 | 0.08-0.8 |
| | | KPS[6] | 0.025 | 0.9 | 0.9-1 |
| | | V-ATPase G1 score (High) | 0.004 | 5.6 | 1.7-16.7 |

[1] The sample size of patients available for each analysis is indicated in brackets
[2] Time of median patients' time to progression or overall survival is expressed in months with 95% CI in brackets
[3] All covariates were categorical. For patients' age, 50 years of age was used as cut-off; for V-ATPaseG1 or Ki67, patients were grouped in low or high expressors according to the protein level established by ROC analysis. For MGMT, patients were categorized in methylated or unmethylated (UM).
[4] HR, Hazard Ratio
[5] CI, Confidence interval
[6] KPS, Karnofsky Performance Score

**Supplementary Table 3.** Clinicopathological features of glioma organotypic tissue cultures.

| GBM culture ID | Gender | Age (y) | Ki67 (%) | MGMT[1] | 1p/19q LOH[2] | IDH1[3] | V-ATPase G1 score[4] | Ki67 score/T0[5] |
|---|---|---|---|---|---|---|---|---|
| OC1 | F | 55 | 70 | UM | Neg | WT | 120/ High expressor | 0.1 |
| OC2 | F | 73 | 40 | M | 1p | WT | 10/ Low expressor | 0.3 |
| OC3 | M | 43 | 30 | UM | 19q | WT | 40/ High expressor | 0.04 |
| OC4 | M | 75 | 15 | M | Neg | np | 30/ High expressor | 0.1 |
| OC5 | M | 39 | 50 | M | np | WT | 30/ High expressor | 0.02 |
| OC6 | M | 65 | 25 | UM | Neg | WT | 22.5/ High expressor | 0.03 |
| OC7 | M | 40 | np | M | 1p/19q | M (R132H) | 2/ Low expressor | 0.1 |
| OC8 | M | 59 | 30 | UM | Neg | WT | 90/ High expressor | 0.03 |

[1] M, Methylated; UM, Unmethylated
[2] Neg, absence of LOH at the indicated loci; np, not performed
[3] WT, Wild Type; M, Mutated; np, not performed
[4] V-ATPase G1 IHC score as determined in uncultured sample
[5] Ki67 as detected in cultures treated with 2μM of BafA1 for 72 hours

**Supplementary Table 4.** Gene expression assays used in the study are indicated with their commercial and Ensemble IDs.

| Gene name | Assay ID | Ensemble ID |
|---|---|---|
| ATP6V0A2 | Hs00429389-m1 | ENSG00000185344 |
| ATP6V0C | Hs00798308-Sh | ENSG00000185883 |
| ATP6V1C1 | Hs00940702_m1 | ENSG00000155097 |
| ATP6V1G1 | Hs00895280_g1 | ENSG00000136888 |
| ATP6V1G2 | Hs00431046_m1 | ENSG00000213760 |
| ATP6V1G3 | Hs00373169_m1 | ENSG00000151418 |
| NESTIN | Hs04187831_g1 | ENSG00000132688 |
| PROM1/CD133 | Hs01009250_m1 | ENSG00000007062 |
| SOX2 | Hs01053049_s1 | ENSG00000181449 |
| POU3F2 | Hs00271595_s1 | ENSG00000184486 |
| SALL2 | Hs00826674_m1 | ENSG00000165821 |
| OLIG2 | Hs00300164_s1 | ENSG00000205927 |

## Supplementary Figures

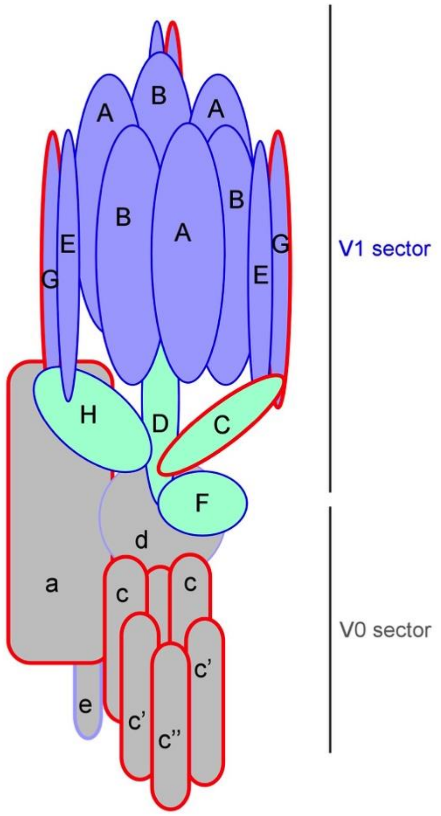


**Supplementary Fig. 1. Schematic of V-ATPase pump.** The subunits of the V0 or V1 sector are colored in grey or blue, respectively. Analyzed subunits are circled in red.

**A**

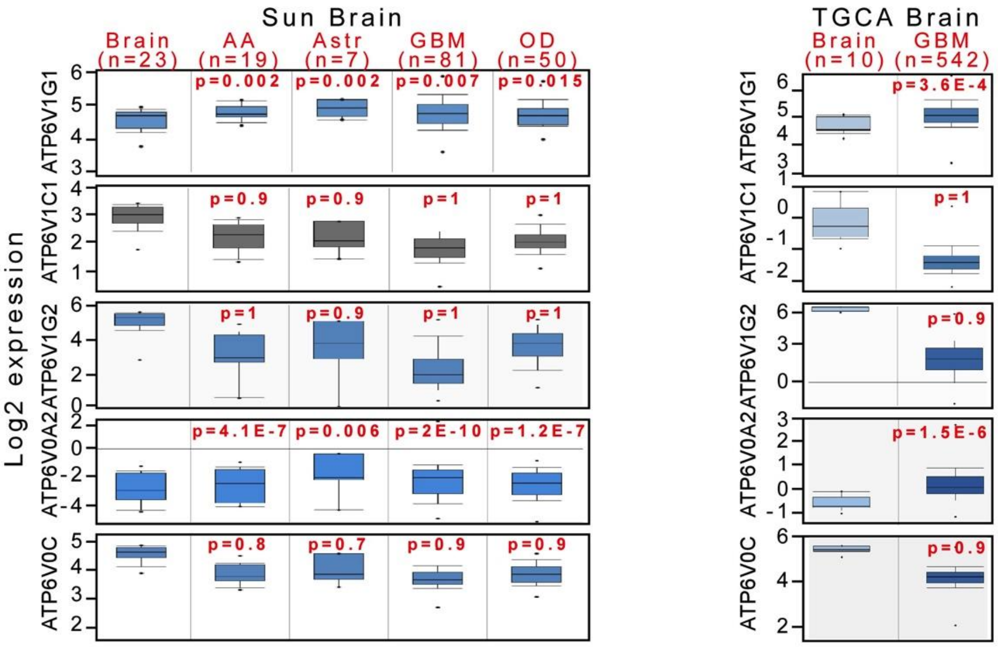


**B**

| | | | ATP6V1G1 overexpression | | ATP6V1C1 overexpression | | | | |
|---|---|---|---|---|---|---|---|---|---|
| DATASET NAME | Samples in the analysis [1] | n | COMPARISON | p-Value (*t* Test) | COMPARISON | p-Value (*t* Test) | Overexpression of other V-ATPase subunits [2] | Co-expression of ATP6V1G1 with other V-ATPase | Co-expression of ATP6V1C1 with other V-ATPase |
| SHAI | BRAIN, Astr, OD, GBM | 42 | NORM vs OD | 5.52E-06 | NORM vs OD | 0.03 | | No | No |
| | | | NORM vs ASTR | 5.37E-04 | NORM vs ASTR | 0.003 | ATP6V1A1, ATP6V1D | | |
| | | | NORM vs GBM | 1.47E-04 | NORM vs GBM | ns | ATP6V1A1 | | |
| | | | OD vs OTHERS | 1.22E-04 | ASTR vs GBM | 0.006 | | | |
| TGCA | BRAIN, GBM | 557 | NORM vs GBM | 3.60E-04 | NORM vs GBM | ns | ATP6V0A2 | No | No |
| FRENCH | BRAIN, AO, AOS | 33 | NORM vs AO | 0.001 | NORM vs AO | ns | ATP6V0A2 | No | ATP6V1H; ATP6V1A |
| | | | NORM vs AOS | 5.90E-04 | NORM vs AOS | ns | ATP6V0A2 | | |
| MURAT | BRAIN, GBM | 84 | NORM vs GBM | 2.54E-11 | NORM vs GBM | ns | ATP6V0A2 | No | No |
| SUN | BRAIN, ASTR, AA, OD, GBM | 180 | NORM vs ASTR | 0.002 | NORM vs ASTR | ns | ATP6V0A2, ATP6V1B1 | No | No |
| | | | NORM vs AA | 0.002 | NORM vs AA | ns | ATP6V0A2, ATP6V1B1 | | |
| | | | NORM vs OD | 0.015 | NORM vs OD | ns | ATP6V0A2, ATP6V1B1 | | |
| | | | NORM vs GBM | 0.007 | NORM vs GBM | ns | ATP6V0A2, ATP6V1B1 | | |

[1] ASTR, Astrocytoma; OD, Oligodendroglioma; AA, Anaplastic astrocytoma; AO, Anaplastic oligodendroglioma; AOS, Anaplastic oligoastrocytoma

[2] p<0.01. Expression of ATP6V1A, ATP6V1B1, ATP6V1D, ATP6V1E1, ATP6V1F, ATP6V1G2, ATP6V1G3, ATP6V1H; ATP6V0A1, ATP6V0A2, and ATP6V0C was analyzed.

**C**

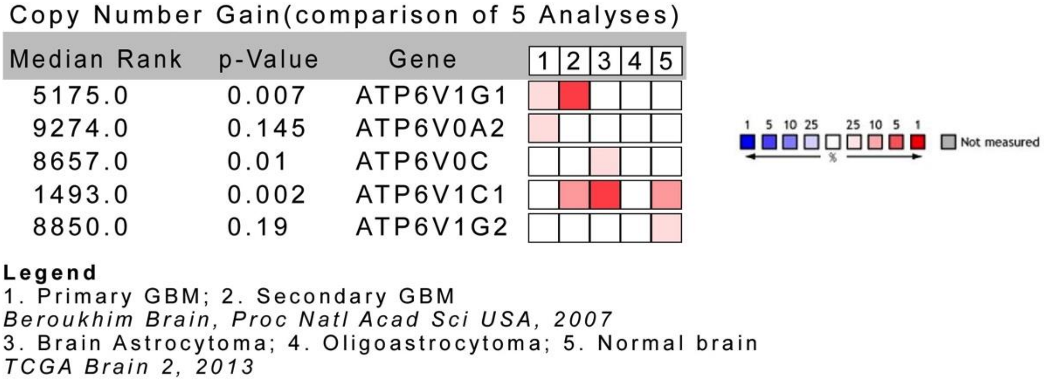


**Supplementary Fig. 2. Oncomine Brain Cancer Microarray database analysis of V-ATPase genes expression profiles**. Public microarray datasets from Oncomine repository were queried for

differential expression of the indicated V-ATPase in gliomas and normal brain tissues (**A, B**). Datasets are reported using the name of the author as it appears in Oncomine repository (ns, not significant). **C**. ATP6V1G1, ATP6V1G2, ATP6V1C1, ATP6V0A2 and V-ATPASEV0C copy number variation was analyzed in brain cancer datasets available in Oncomine repository. Red box, significant copy number gain.

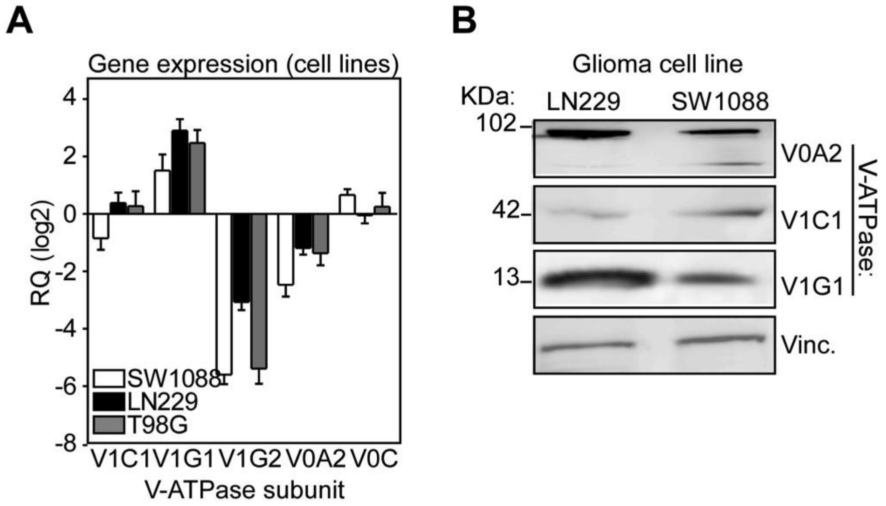


**Supplementary Fig. 3. V-ATPase subunits expression in commercial glioma cell lines. A**) The indicated V-ATPase subunit was analyzed in astrocytoma (SW1088) or glioblastoma (LN229 and T98G) cell lines by qPCR. **B**) V-ATPase subunits V0A2, V1C1 and V1G1 expression was analyzed by immunoblotting in SW1088 and LN229 cells.

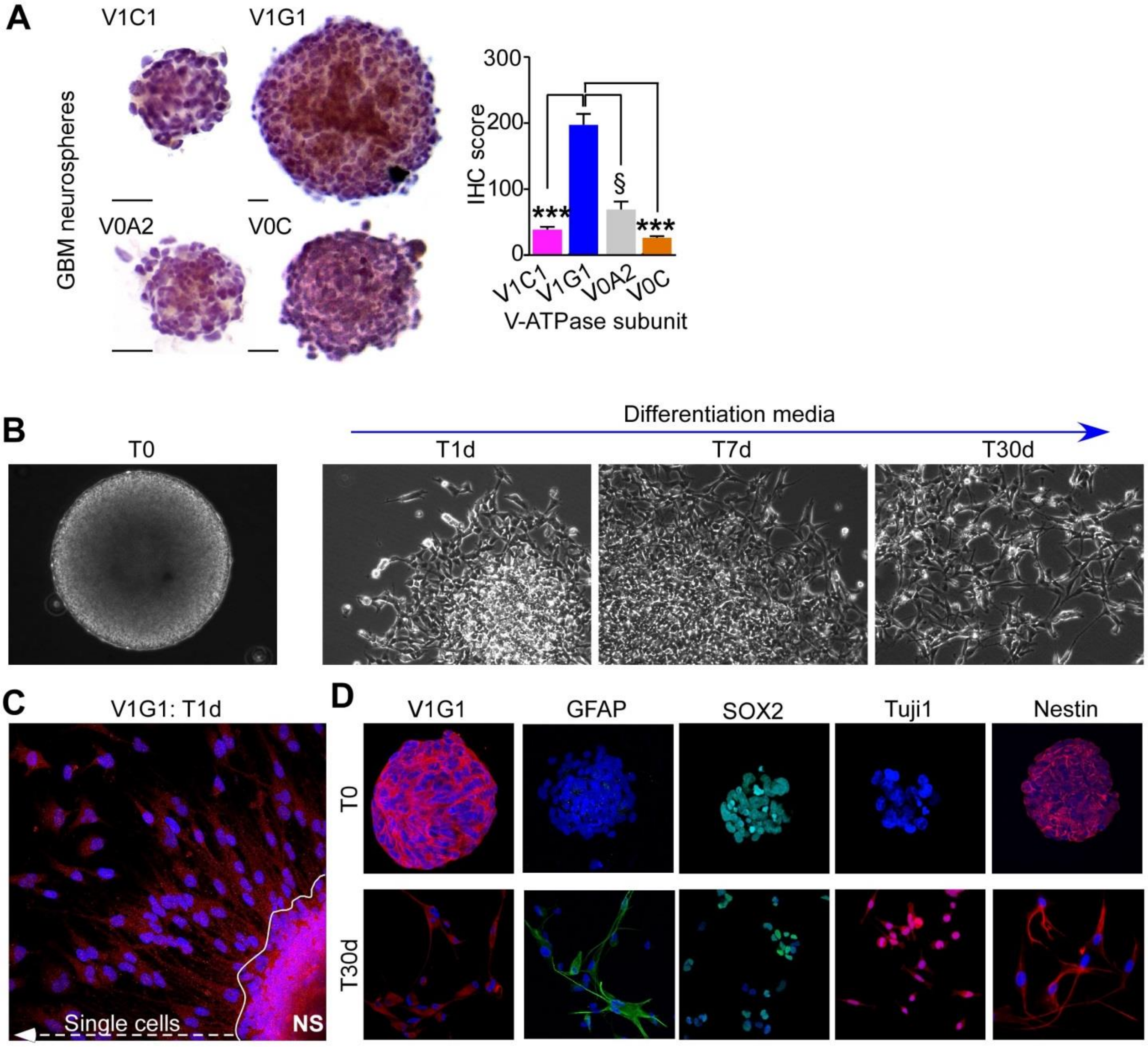


**Supplementary Fig. 4. V-ATPase G1 expression is enriched in neurospheres**. **A**) Analysis of V-ATPase subunits expression in GBM neurospheres by immunohostochemistry. Representative pictures of V1C1, V1G1, V0A2 and V0C subunits are shown. Scale bar represents 100µm. *Right*, quantification of V-ATPase subunits IHC scores (n=10 neurospheres per protein). ***, p=0.0001; $^{§}$, p=0.0002 by Mann-Whitney U test. Bars, mean±SEM. **B-D**) Neurospheres cultivated in a serum-based media differentiated into monolayers cell cultures over a period of 30 days. Expression of V-ATPase G1 (F, G) or immunophenotypic markers was assessed after 1 day of differentiating growth condition (D) or after 30 days (T30d, E) and compared to that of primary neurospheres (T0). Representative maximum intensity projection images are shown for stem-cell (Nestin, Sox2) or

differentiation (GFAP, Tuji1/βIII Tubulin) markers. DAPI stained nuclei. NS, neurosphere. Original magnification x400.

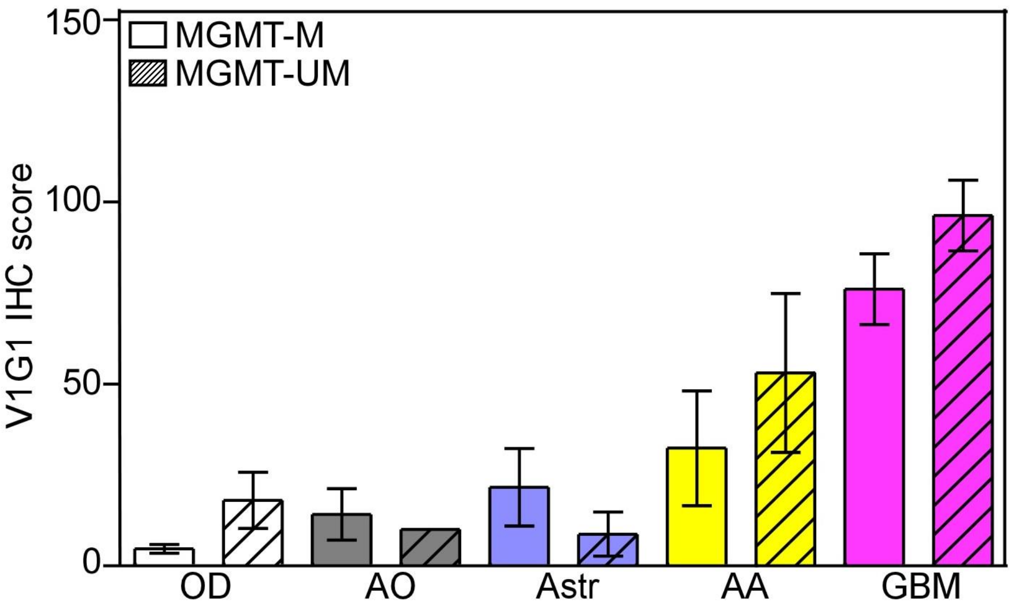


**Supplementary Fig. 5. Correlation of V-ATPase subunit G1 expression with MGMT status**. The immunoreactivity of V-ATPase subunit G1 according to MGMT promoter methylation is reported for all gliomas. Striped bars, unmethylated samples (UM); full bars, methylated samples (M). OD, oligodendroglioma; AO, anaplastic OD; Astr, astrocytoma; AA, anaplastic Astr; GBM, glioblastoma. Bars represent mean±SEM.

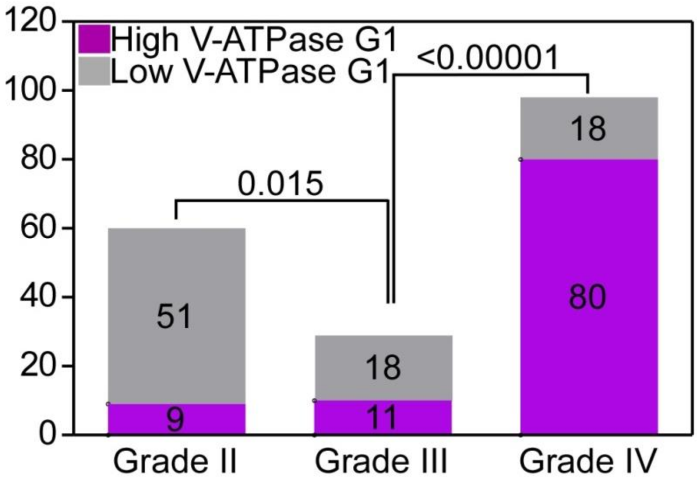


**Supplementary Fig. 6.** V-ATPase subunit G1 expression correctly classifies brain tumors according to the histological grade. The number of grade II, III or IV (GBM) patients with high (IHC score >21.5) or low V-ATPase G1 expression, as established by the ROC curve (see Figure 2D), is illustrated. *P* values are from chi-square test.

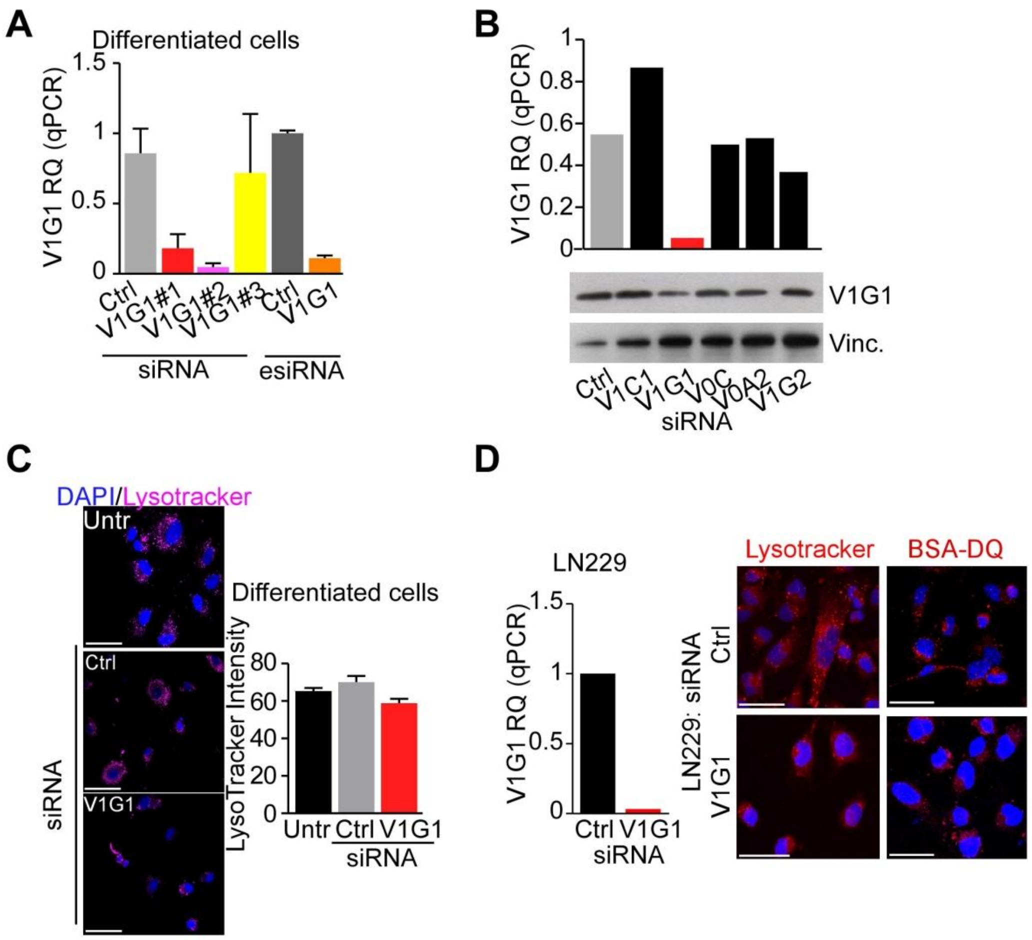


**Supplementary Fig. 7**. **ATP6V1G1 siRNA in differentiated GBM cultures or LN229 cells**. **A)** Quantification of ATP6V1G1 mRNA expression in differentiated primary GBM cell cultures after the indicated siRNA or esiRNA. For subsequent siRNA experiments the V1G1#1 molecule was used. **B**) ATP6V1G1 gene (qPCR; *top panel*) or protein (western-blot; *bottom panel*) levels in differentiated GBM cultures after the indicated siRNA. **C**) Confocal immunofluorescence analysis of live GBM monolayers transfected with a non-targeting (Ctrl) or a ATP6V1G1-directed siRNA (V1G1) or left untreated (Untr) for 48 hours and then incubated with LysoTracker Red for 10 minutes at 37°C. Original magnification x 400. *Left*, quantification of mean fluorescence intensity (n=5). **D**) Live LN229 treated as in **C** were evaluated by confocal analysis for incorporation of either LysoTracker Red or DQ-BSA probes.

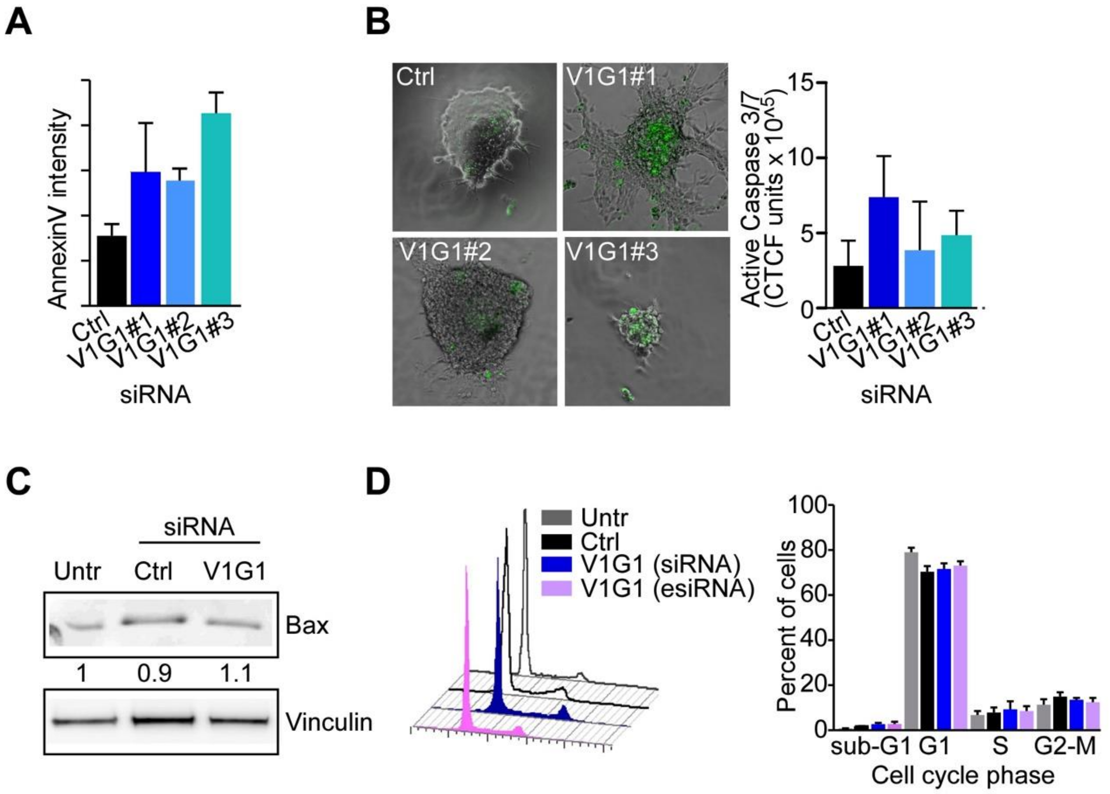


**Supplementary Fig. 8. Targeting of V1G1 by transient gene knockdown activates caspase 3/7 in neurospheres. A, B**) Apoptosis induction after ATP6V1G1 knockdown in patients' derived neurospheres using three different siRNAs (V1G1#1,2,3 siRNA) was evaluated by Annexin V staining and fluorescence microscopy (**A**) or activation of effector caspases 3/7 (**B**). Bars, quantification of mean fluorescence intensity ±SEM (n=5). CTCF, Corrected total cell fluorescence. **C**) The pro-apoptotic Bcl2 family member Bax is not increased after V1G1 knockdown. **D**) Cell cycle phases' transition in neurospheres treated with the indicated siRNA or esiRNA. *Right*, quantification of cells in cycle phases. Bars, mean ±SEM (n=3).

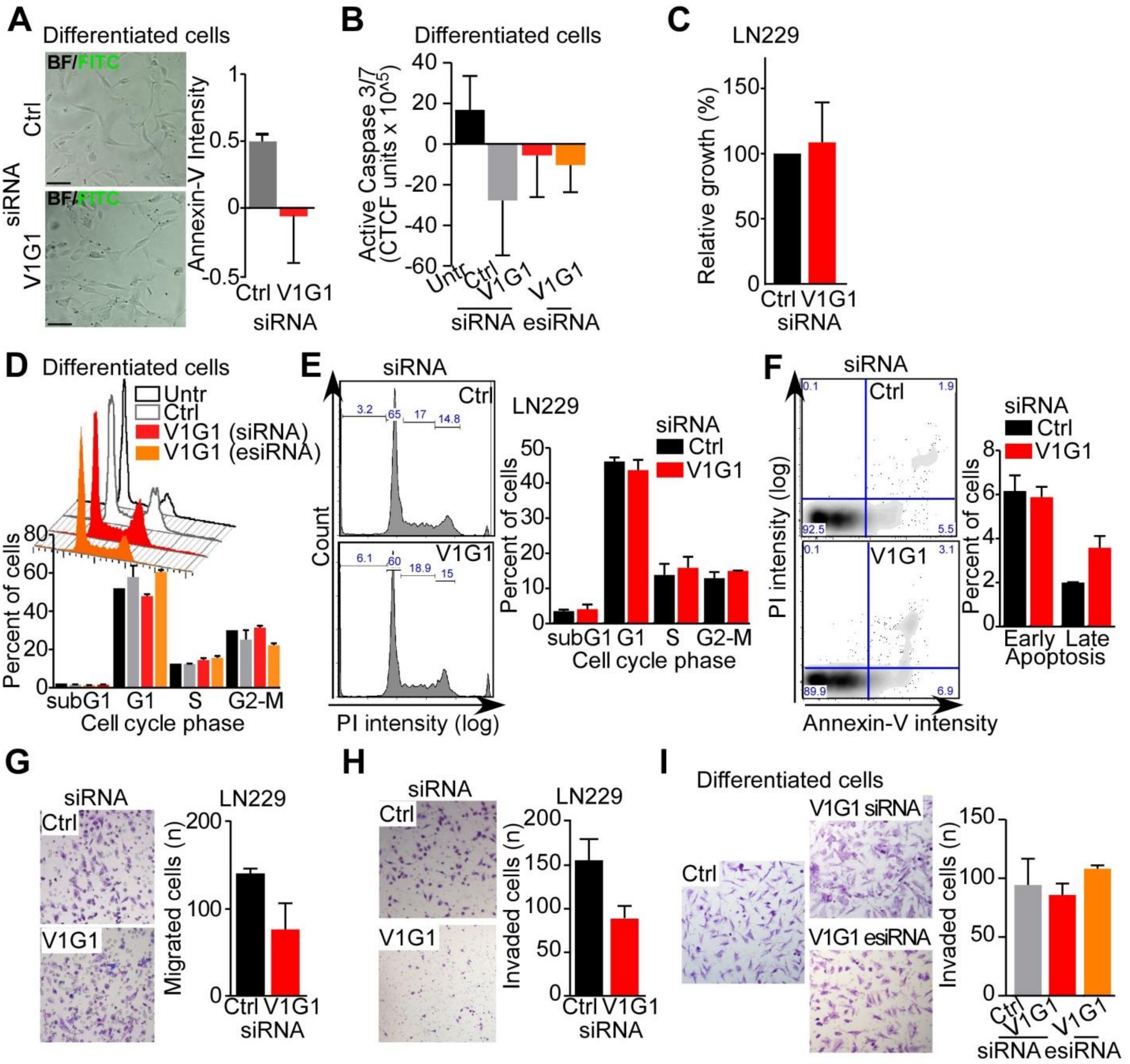


**Supplementary Fig. 9. Targeting of V-ATPase G1 by gene knock-down does not impair differentiated tumor cultures viability and motility. A,B**) Apoptosis induction after ATP6V1G1 knockdown (V1G1 siRNA or esiRNA) was evaluated in GBM differentiated cells by Annexin V staining and fluorescence microscopy (**A**) or by active caspases 3/7 detection and fluorescence microscopy (**B**). *Right,* Quantification of mean fluorescence intensity (n=3). Scale bar indicates 10µm. CTCF, Corrected total cell fluorescence. **C)** MTT assay was performed in LN229 after transfection with the V1G1#1 or a control (Ctrl) siRNA. Relative cell growth was calculated assuming the control sample was 100%. **D-F**) Cell cycle phases transition (PI staining, **D,E**) or

apoptosis (**F**, Annexin-V staining) was evaluated by fluorescence-assisted flow cytometry analysis in differentiated GBM monolayers or LN229 cells treated as before. Early or late apoptosis (**F**) was defined as single FITC-positive or double FITC-PI positive cells respectively. **G-I**) GBM cell migration (LN229, **G**) or invasion (LN229 or GBM differentiated cells; **H, I**) was evaluated by trans-well assays coated with collagen or matrigel, respectively, and quantified by direct cell counting. *Left*, representative photomicrograph of the indicated cells. Original magnification x 100.

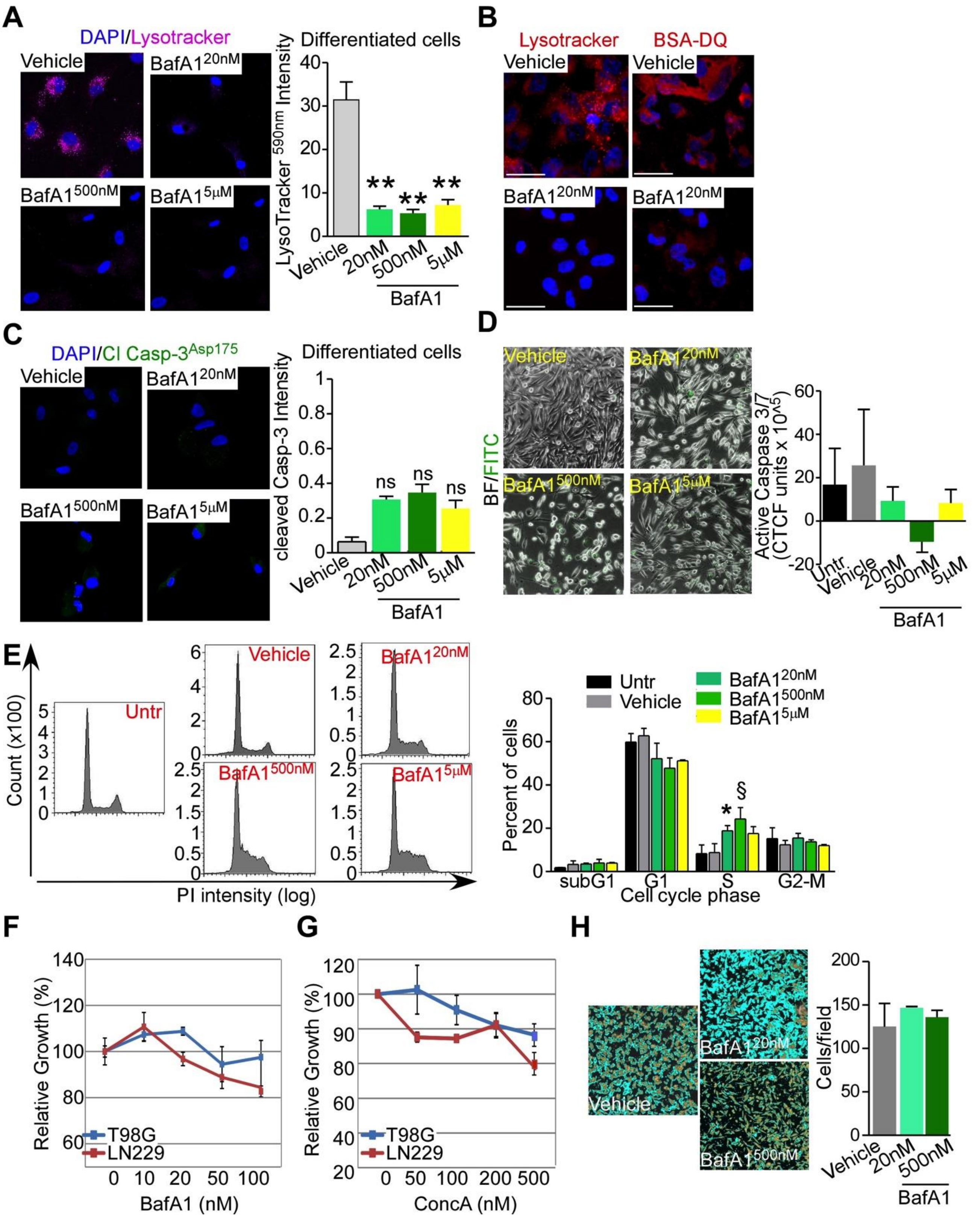


**Supplementary Fig. 10. Effects of V-ATPase pump activity inhibition by selective compounds in GBM differentiated cells or LN229 cultures**. **A,B**) Confocal immunofluorescence analysis of live GBM monolayers (**A**) or LN229 cells (**B**) treated with vehicle, or the indicated concentrations

of bafilomycinA1 (BafA1) for 48 hours and then incubated with LysoTracker Red$^{590}$ or DQ-BSA probes for 1 h at 37°C. Maximum intensity projection images are shown for a representative experiment. Original magnification x 400. *Right*, quantification of mean fluorescence intensities (n=5 fields for three independent experiments). **C,D)** Cleavage of Caspase-3 at Asp175 residue (**C**) or activation of caspases 3/7 (**D**) in GBM differentiated cell cultures treated with the indicated concentration of BafA1 or vehicle was investigated by immunostaining or fluorescence microscopy, respectively. *Right,* Quantification of mean fluorescence intensity (n=5 fields for three independent experiments). Original magnification x 400. **E**) Cell cycling of GBM differentiated cells treated with the indicated concentration of bafilomycin A1, vehicle, or left untreated (Untr), was analyzed by PI staining and flow cytometry. *Right*, quantification of cells subpopulations in cell cycle phases subG1, G1, S and G2-M. *, p=0.014; §, p=0-004 compared to vehicle by Mann-Whitney U test by **F,G**) MTT assays were performed in LN229 treated with different concentrations of bafilomycinA1 (BafA1, **D**) or concanamycin A (ConcA, **E**) for 48 hours. Relative cell growth was calculated assuming the control sample was 100%. **H**) Invasion of differentiated GBM cell in presence or absence of bafilomycin A1 was evaluated by trans-well assays coated with matrigel and quantified by direct cell counting. *Left*, representative photomicrograph of the indicated cells. Original magnification x 100.
